# The strategy dynamics of collective systems: Underlying hindrances beyond two-actor coordination

**DOI:** 10.1371/journal.pone.0301394

**Published:** 2024-04-01

**Authors:** Ambrosio Valencia-Romero, Paul T. Grogan

**Affiliations:** 1 The Roux Institute at Northeastern University, Portland, ME, United States of America; 2 School of Computing and Augmented Intelligence, Arizona State University, Tempe, AZ, United States of America; Southwest University, CHINA

## Abstract

Engineering systems, characterized by their high technical complexity and societal intricacies, require a strategic design approach to navigate multifaceted challenges. Understanding the circumstances that affect strategic action in these systems is crucial for managing complex real-world challenges. These challenges go beyond localized coordination issues and encompass intricate dynamics, requiring a deep understanding of the underlying structures impacting strategic behaviors, the interactions between subsystems, and the conflicting needs and expectations of diverse actors. Traditional optimization and game-theoretic approaches to guide individual and collective decisions need adaptation to capture the complexities of these design ecosystems, particularly in the face of increasing numbers of decision-makers and various interconnections between them. This paper presents a framework for studying strategic decision-making processes in collective systems. It tackles the combinatorial complexity and interdependencies inherent in large-scale systems by representing strategic decision-making processes as binary normal-form games, then dissects and reinterprets them in terms of multiple compact games characterized by two real-numbered structural factors and classifies them across four strategy dynamical domains associated with different stability conditions. We provide a mathematical characterization and visual representation of emergent strategy dynamics in games with three or more actors intended to facilitate its implementation by researchers and practitioners and elicit new perspectives on design and management for optimizing systems-of-systems performance. We conclude this paper with a discussion of the opportunities and challenges of adopting this framework within and beyond the context of engineering systems.

## 1 Introduction

Understanding emergence in large-scale systems-of-systems has become a prominent feature in addressing complex real-world challenges [1]. Such collective systems entail the integration of interconnected entities, each with a level of operational and managerial autonomy from the others, while collectively contributing to the overarching system’s objectives. Collaborative decision-making plays a pivotal role in facilitating information exchange and coordinating the activities of individual actors to guide the system toward optimized performance. However, despite the potential benefits of coordinated collective action, its seamless execution faces structural challenges related to the combinatorial complexity of aligning individual objectives and incentives [2]. These hindrances go beyond localized actor-to-actor coordination challenges and encompass intricate dynamics that result from the interplay of many subsystems, direct and indirect stakeholders, and decision-making agents. Addressing these challenges necessitates a deep understanding of the underlying structure of the collective decision-making process that impacts the strategic behaviors and interactions within the system.

Engineering systems are a subset of collective systems marked by high technical complexity, social intricacy, and elaborate processes geared toward fulfilling significant societal functions [3]. While general collective systems refer to groups of agents collaborating to produce a collective behavior that transcends the sum of individual contributions [4], the nuanced nature of engineering systems implies the need for a strategic design approach to navigate their multifaceted challenges. The complexity within engineering systems becomes evident in endeavors such as aircraft design, where societal needs and expectations, communicated through regulations, can directly impact lower-level technical decisions [5]. The evolving concept of complexity within engineering systems necessitates ongoing efforts to define, measure, and comprehend its intricate nature. Challenges arise in understanding and modeling emergence, a fundamental characteristic of complex collective systems, which manifests in various forms within engineering contexts, such as non-linear emergent properties and those arising from interdependent choices in multi-agent systems [6].

The strategic design of engineering systems seeks to tackle the complex interplay between technical and social dimensions. In the technical realm, the challenge lies in comprehending interactions between subsystems while maintaining their levels of operational and managerial independence [7]. Simultaneously, the social dimension of engineering systems involves diverse actors, categorized into users, stakeholders, and societal entities. The conflicting needs and expectations of these actors amplify the complexity of the system design [3]. The strategic challenges embedded in engineering systems design, particularly concerning the emergence of unfavorable dynamics and the modeling of interactive decisions among multiple actors, highlight the need for advanced methodologies. Traditional game-theoretic approaches, often designed for two-player scenarios, must be revised to capture the intricacies of complex design ecosystems [5]. The classification of emergence based on non-linearities and multi-agent systems guides the selection of appropriate methods for system-level governance, necessitating shifts in design perspectives [6]. As engineering systems become more complex, a comprehensive and adaptable framework is increasingly necessary to address strategic challenges in their intricate design landscapes.

Game theory is a valuable framework to address the strategic challenges embedded in engineering systems. As the mathematical study of strategic interactions among independent actors, the last four decades have seen an increasing number of researchers employing game-theoretical methods to model decision dynamics in multidisciplinary engineering systems, treating different systems actors as distinct players engaged in a sequence of games throughout the design process [8–10]. Recent literature shows applications of game theory to study modularity as a mechanism for facilitating cooperation in systems-of-systems [11], develop computational models to assist engineering systems design under competition [12], and investigate the link between risk attitudes and strategic decisions in the context of collective systems [13]. Existing formulations emphasize dynamics among two actors, for which extensive work exists centered on Nash equilibria in social dilemma games. However, as design ecosystems become more intricate, existing game-theoretic approaches need to be revised in modeling the complex interactions among an increasing number of design actors [5]. Broader classes of engineered systems—including inter-agency, international, or public-private partnerships—exhibit strategy dynamics among more than two actors. More existing work is needed to formulate and understand the emergent dynamics in strategic scenarios with three or more actors due partly to a combinatorial growth of possible outcomes.

We turn to non-cooperative game theory to comprehend the nature of these strategy dynamics in collective systems. By adopting this analytical lens, we gain valuable insights into the strategic components associated with the stability of collective action and the risk of coordination failure among multiple decision-makers pursuing their self-interests. The application of game theory in this context helps us analyze how individual motivations and incentives can influence collaborative decision-making processes and how this, in turn, impacts the system’s overall performance and collective efficiency. Understanding and describing the interdependence of decisions among the actors in the system is essential to producing useful models of emergent behavior and anticipating potential outcomes that cannot be solely attributed to individual components. By investigating these dynamics, we can shed light on how collective systems adapt and respond to various stimuli, offering new perspectives on system design and management.

### 1.1 Motivating challenges

Understanding the emergence of strategies within complex collective systems poses significant challenges for engineers and system designers when evaluating a system’s ability to integrate with others and align with common goals. This property, referred to as directional integrability [14], when extended to larger systems-of-systems, is linked to each subsystem’s level of managerial control and collaboration [7]. Individual actors within the system face critical choices regarding the openness of their subsystems, and these choices have far-reaching implications for the system’s overall performance. The decision to pursue an open strategy, facilitating full integration, or to maintain a closed system introduces complex trade-offs. Actors must weigh the potential benefits of collective integration against the costs and risks associated with opening their own systems. Although an open strategy might be optimal on a collective level, it might not be the most advantageous path for the individual actor.

The complexity escalates dramatically as the number of actors within the system increases. Decision-making becomes a tangled web of anticipating other actors’ choices, potential coalitions, and the risks associated with incomplete information. Understanding the social dynamics at play becomes essential, as factors like communication, the way options are framed [15, 16], and actors’ varying motivations and incentives [17] significantly influence the strategic landscape. A particularly worrying outcome in multi-actor systems is the potential for integration to halt. Imagine a scenario where a single actor initially takes the risk of opening their system, hoping others will follow. However, if others exploit this openness, keeping their systems closed and reaping the benefits without contributing, this can discourage further integration efforts. The system can easily become stuck in a situation where the benefits of wider integration seem out of reach, as no individual actor wants to be the only one taking on the burden of an open system.

To overcome these challenges, there’s a critical need for new modeling approaches and analytical tools explicitly designed to handle the emergent dynamics of multi-actor scenarios. These models must move beyond traditional two-player game-theoretic frameworks to robustly capture complex interactions and coalition formation. Holistic decision-making approaches considering the technical, social, and strategic interdependencies are needed. Alongside sophisticated modeling, a focus on developing mechanisms to incentivize integration is paramount. Finding ways to align individual actor benefits with the goals of the collective system is essential to ensure sustained progress and avoid stagnation. Addressing these challenges is crucial for successfully engineering and managing complex systems-of-systems. A deep understanding of these emergent strategic dynamics and incentive structures will empower us to build systems that foster coordination, reduce incompatibilities, and ultimately facilitate the harmonious integration of interdependent subsystems for the greater good.

### 1.2 Our research endeavor

Our work focuses on studying complex strategy dynamics in collective systems, with an aim to comprehend the factors that influence the decisions and interactions of system actors. We acknowledge the complexity of these systems and strive to design mechanisms that promote collaboration in large-scale systems-of-systems. The objective of this paper is to provide a general mathematical characterization and visual representation of emergent strategy dynamics in binary games that can be leveraged in the strategic design of engineering systems. It extends the existing concept of structural fear and greed in two-player games to any finite number of actors. These structural factors are presented on a Cartesian coordinate plane, with each quadrant defining a dynamical domain with specific strategic stability conditions.

The remainder of this paper is organized as follows. Section 2 recaps the mainstream treatment of strategy dynamics centered around normal-form games with two players and two available strategies each. Section 3 presents our extended characterization of strategy dynamics for binary strategic-form games with any number of players, with more details provided in the S1 Appendix. Here we introduce the concept of *strategic hindrance*, with Section 3.3 applying the proposed framework to assess the evolution of strategy dynamics in the design for integrability of an urban transit system stylized as a volunteer’s dilemma, and Section A.4 in S1 Appendix extending this framework to visually assess additional generic strategic settings (viz. public goods game, fated truel game, majority game, and others). Then, in Section 4, we address the assumptions and limitations of our proposed approach. We conclude with Section 5, summarizing contributions and our vision for future work.

## 2 Strategy dynamics in 2 × 2 social dilemmas

The concept of fear and greed strategy dynamics is based on the study of social dilemmas using game theory. A “game” is any decision-making process involving multiple independent actors with specific preferences over the decisions any of them could make and their consequences. We focus on normal-form games, the most common representations used to model social dilemmas.

### 2.1 Normal-form games

A normal-form game consists of

- A player set N consisting of *n* ≥ 1 player names or other identifiers (ids);
  - for the sake of simplicity, we use N={1,…,n} and dummy ids i,j,k∈N;
  - a game with *n* = 1 is referred to as a “trivial” decision problem.
- A finite set of *pure* individual strategies $S_{i}=\{s_{i}^{\left(1\right)},\;\ldots ,\;s_{i}^{\left(m_{i}\right)}\}∋s_{i}$, with $m_{i}\geq 2\forall i\in N$;
  - a pure strategy is a complete contingency plan that a player can execute in anticipation of the actions that the other players could take;
  - the product $S=S_{1}\times \ldots \times S_{n}$ is the set of all possible pure collective strategies *s* = 〈*s*_1_, …, *s*_*n*_〉, with $|S|=\prod_{i}^{n}m_{i}$;
  - an *n* × 2 normal-form game (also known as *binary* game) is one in which *m*_*i*_ = 2 for every i∈N, resulting in a total of 2^*n*^ pure collective strategies.
- A *payoff* function $U_{i}:S\mapsto R^{n}$ valuing the preference of player *i* for each s∈S;
  - the value of *U*_*i*_(*s*) is assumed to be measured on a cardinal utility scale;
  - $\exists \;s\in S:\;U_{i}(s)\neq 0$, or equivalently, max_*s*_|*U*_*i*_(*s*)| ≠ 0 for every i∈N.

The central objective of game theory is to understand what will, could, or should happen in a given game. One of the main solution concepts in game theory is the Nash equilibrium, which unified several earlier ideas related to the stability of collective action in non-cooperative games [18]. The ideas we develop in this work are relevant to studying pure-strategy Nash equilibria (PNE), in which every player is expected to commit fully to one and only one pure individual strategy (see Definition A.1 in the S1 Appendix).

**Fig 1:**
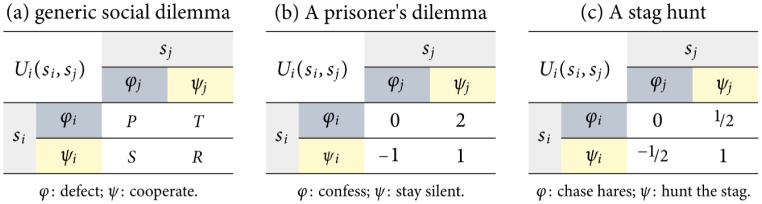
Generic individual payoff matrix in a two-player two-strategy social dilemma game in normal form. The strategy labels *φ* and *ψ* represent “defection” and “cooperation”, respectively. The value of *P* represents the punishment resulting from mutual defection; *R* > *P* is the reward of mutual cooperation; *S* stands for sucker’s payoff (what a player gets from cooperating if the other defects); and *T* stands for temptation to defect (the incentive to deviate away from the cooperative strategy in the expectation of higher returns).

Fig 1(a) and 1(b) show examples of individual payoff matrices in 2 × 2 social dilemmas. We can also write all payoffs in a 2 × 2 game as a simplified bimatrix:

$$\begin{array}{c} {}[\left\langle \;U_{i}\left(s_{i},s_{j}\right)\;,\;U_{j}\left(s_{j},s_{i}\right)\;\right\rangle {]}_{2\times 2}=[\;\begin{array}{cc} \left\langle \;U_{i}\left(\;\;\varphi_{i},\varphi_{j}\right)\;,\;U_{j}\left(\varphi_{j},\;\;\varphi_{i}\right)\;\right\rangle & \left\langle \;U_{i}\left(\;\;\varphi_{i},\psi_{j}\right)\;,\;U_{j}\left(\psi_{j},\;\;\varphi_{i}\right)\;\right\rangle \\ \left\langle \;U_{i}\left(\psi_{i},\varphi_{j}\right)\;,\;U_{j}\left(\varphi_{j},\psi_{i}\right)\;\right\rangle & \left\langle \;U_{i}\left(\psi_{i},\psi_{j}\right)\;,\;U_{j}\left(\psi_{j},\psi_{i}\right)\;\right\rangle \end{array}\;], \end{array}$$ (1)

where $S_{k}=\{\varphi_{k},\psi_{k}\}$ for every player k∈N={i,j}. The collective strategy set is $S=S_{i}\times S_{j}=\{\langle \varphi_{i},\varphi_{j}\rangle ,\langle \varphi_{i},\psi_{j}\rangle ,\langle \psi_{i},\varphi_{j}\rangle ,\langle \psi_{i},\psi_{j}\rangle \}$. Strategies *φ* = 〈*φ*_*i*_, *φ*_*j*_〉 and *ψ* = 〈*ψ*_*i*_, *ψ*_*j*_〉 are called *diagonal*; the other collective strategies, namely those in set S\{φ,ψ}, are called *off-diagonal*. Labeling a combination of individual strategies as diagonal or off-diagonal is left to the reader’s discretion, and it does not affect the nature of the game. In this work, *φ* is also referred to as the *status quo* strategy—or starting point—and *ψ* is treated as the alternative or *desired* outcome (from the perspective of the incentive designer or researcher) consistent with economics literature [19].

### 2.2 Structural fear and greed in 2 × 2 social dilemmas

Classical 2 × 2 social dilemmas name each strategy after the description that human players would use to describe their behavior [15] and are often symmetric—meaning their payoff functions do not depend on the players’ identities, i.e. $U_{i}(s_{i}^{\prime },s_{j})=U_{j}(s_{j}^{\prime },s_{i})$. For instance, let us assume that *φ*_*k*_ is a “defective” individual strategy and that *ψ*_*k*_ is a “cooperative” one. Coordinating on *φ* and *ψ* can thus be called mutual (or unanimous) defection and cooperation, respectively. These are the usual strategy labels in a classical social dilemma. Fig 1(a) shows a generic payoff matrix used to characterize these games. Cooperation yields a “reward”, *U*_*i*_(*ψ*_*i*_, *ψ*_*j*_) = *R*, which would be preferable to mutual defection, which results in a “punishment”, *U*_*i*_(*φ*_*i*_, *φ*_*j*_) = *P* < *R* [20]. The values *U*_*i*_(*ψ*_*i*_, *φ*_*j*_) = *S* and *U*_*i*_(*φ*_*i*_, *ψ*_*j*_) = *T* are called “sucker’s” and “temptation” payoffs, respectively, terms borrowed from the *prisoner’s dilemma* (PD) game [21]. A sample individual payoff matrix of a PD game is shown in Fig 1(b). A PD game is characterized by the relationships *T* > *R* > *P* > *S*. The *stag hunt* game, in contrast, of which a sample individual payoff matrix is presented in Fig 1(c), satisfies *R* > *T* > *P* > *S* [22]—a “periodic table” of 2 × 2 games can be derived from different orderings of *P*, *R*, *S*, and *T* as described in Ref. [23].

The *S* payoff is associated with a player’s “fear” of pursuing cooperation when others might defect. Meanwhile, the *T* payoff is a player’s incentive to defect away from mutual cooperation driven by the “greed” for higher returns. Ahn et al. [24] measure structural fear and greed, respectively, as

$$F_{i}\left(\varphi ,\psi \right)=\frac{P-S}{\text{max}\left\{P,R,S,T\right\}-\text{min}\left\{P,R,S,T\right\}}\;\in \;[-1,+1]$$ (2)

and

$$G_{i}\left(\varphi ,\psi \right)=\frac{T-R}{\text{max}\left\{P,R,S,T\right\}-\text{min}\left\{P,R,S,T\right\}}\;\in \;[-1,+1].$$ (3)

The expressions “*P* − *S*” and “*T* − *R*” in Eqs (2) and (3) are the deviation losses measured with respect to the status quo strategy *φ*_*i*_. In the context of the 2 × 2 PD game, the structural fear (*F*_*i*_ > 0) is the deficit of unilaterally cooperating when the other defects and the structural greed (*G*_*i*_ > 0) is the benefit of taking advantage of a cooperating partner by choosing to defect instead [25]. Either factor favors defection if positive. The higher the values of *F*_*i*_ and *G*_*i*_, the greater the incentive that player *i* has to defect instead of playing a cooperative strategy.

Previous works have identified four 2 × 2 strategy dynamical domains exhibiting different payoff and risk dominance conditions based on the values of *S* and *T*, while setting *P* = 0 and *R* = 1 [26–29]. These values map to one combination of positive (high) or negative (low) structural fear and greed, as provided below and in Fig 1, per Eqs (2) and (3):

Harmony / HA (or cooperation):

$$\begin{array}{cc} S>0\;\;\;\wedge \;\;\;T<1 & \;\Leftrightarrow \;F_{i}<0\;\;\;\wedge \;\;\;G_{i}<0 \end{array}$$

Coexistence / CX (or anti-coordination):

$$\begin{array}{cc} S>0\;\;\;\wedge \;\;\;T>1 & \;\Leftrightarrow \;F_{i}<0\;\;\;\wedge \;\;\;G_{i}>0 \end{array}$$

Bistability / BI (or coordination):

$$\begin{array}{cc} S<0\;\;\;\wedge \;\;\;T<1 & \;\Leftrightarrow \;F_{i}>0\;\;\;\wedge \;\;\;G_{i}<0 \end{array}$$

Defection / DE:

$$\begin{array}{cc} S<0\;\;\;\wedge \;\;\;T>1 & \;\Leftrightarrow \;F_{i}>0\;\;\;\wedge \;\;\;G_{i}>0 \end{array}$$

We introduce a fifth dynamical domain to represent player *i*’s indifference about the strategy set: Indifference / ZZ:

$$\begin{array}{cc} S=0\;\;\;\wedge \;\;\;T=1 & \;\Leftrightarrow \;F_{i}=0\;\;\;\wedge \;\;\;G_{i}=0 \end{array}$$

The 2 × 2 harmony and defection dynamical domains are also known as (type *ψ* or type *φ*) *dominance* dynamics [30, 31]; while the coexistence and bistability domains are sometimes referred to as *negative* and *positive frequency dependence dynamics*, respectively [32, 33]. The latter are also known as *bipolar* [34]—note that any 2 × 2 coexistence game can be transformed into a bistability/bipolar game by switching the strategy labels of one of the two players.

**Fig 2:**
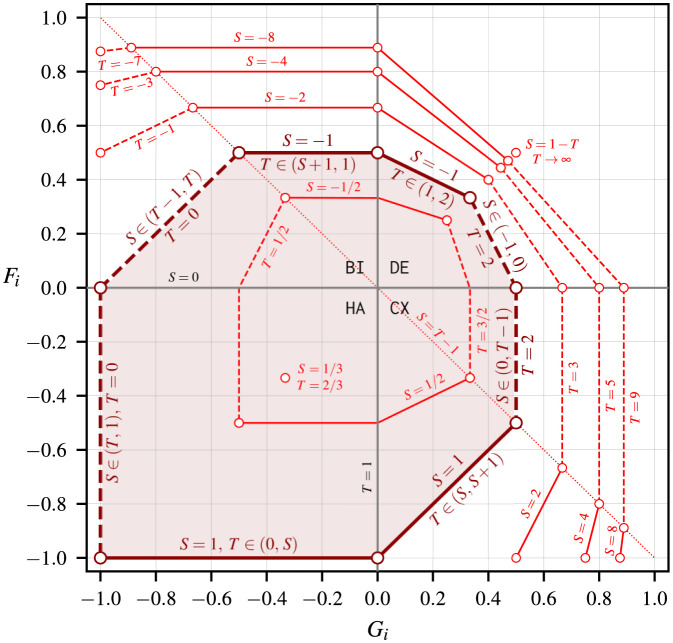
Mapping between the sucker’s (*S*) and the temptation (*T*) payoffs and the structural fear (*F*_*i*_) and structural greed (*G*_*i*_) factors per Eqs (2) and (3), assuming *P* = 0 and *R* = 1. Each quadrant is labeled after the 2 × 2 strategy dynamics resulting from different combinations of low and high levels of structural fear and greed: HA / harmony (*F*_*i*_ < 0, *G*_*i*_ < 0); CX / coexistence (*F*_*i*_ < 0, *G*_*i*_ > 0); BI / bistability (*F*_*i*_ > 0, *G*_*i*_ < 0); and DE / defection (*F*_*i*_ < 0, *G*_*i*_ > 0). The vast majority of the literature on social dilemma games focuses on the shaded region defined by *S* ∈ [−1, 1] and *T* ∈ [0, 2].

Fig 2 shows how the values of *S* and *T* map to the factors *F*_*i*_ and *G*_*i*_ per Eqs (2) and (3), assuming *P* = 0 and *R* = 1. The vast majority of the literature on social dilemma games focuses on the payoff region defined by *S* ∈ [−1, 1] and *T* ∈ [0, 2]. These games cover the entire harmony domain, ⅜ of the bistability and coexistence domains, and only ⅙ of the defection domain—that is, 2348 of the whole fear and greed strategy dynamics space. The structural fear and greed values for the PD game in Fig 1(a) are *F*_*i*_ = *G*_*i*_ = 1/3, which fall on defection dynamics, while the values for the SH game in Fig 1(b) are *F*_*i*_ = 1/3 and *G*_*i*_ = −1/3, falling on bistability dynamics. Examples of harmony and coexistence games respectively include the *concord* game (*S* = 1/3 and *T* = 2/3 ⇒ *F*_*i*_ = *G*_*i*_ = −1/3) and the *chicken* game (*S* = 1/2 and *T* = 3/2 ⇒ *F*_*i*_ = −1/3 and *G*_*i*_ = 1/3).

### 2.3 Deviation losses and strategic stability

For general *n* × 2 games, let us define the *rescaled deviation loss* *ℓ*_*i*_ in terms of the difference in player *i*’s payoff when deviating from $s_{i}^{*}$ to $s_{i}\in S_{i}$ assuming the strategies of the other players remain fixed:

**Definition 1** *Let* i∈N *be a* standpoint *player in the n-player normal-form game*

$$G_{N}=(\;N,(S_{i}{)}_{i\in N},(U_{i}{)}_{i\in N}\;),$$

*where* N *is the set of n players*, $S_{i}$ *is the set of individual strategies*, $S=S_{i}^{n}$ *are the collective strategies, and* $U_{i}:S\mapsto R$ *is player i’s payoff function. The rescaled individual payoff loss of deviating from* $s_{i}^{*}$ *to* $s_{i}\in S_{i}$ *contingent on a collective strategy s*_−*i*_ *played by all other players* (−i=N\{i}) *is*

$$\begin{array}{c} \ell_{i}\left(s_{i}^{*},s_{-i}\right)=\frac{1}{A_{i}}[U_{i}\left(s_{i}^{*},s_{-i}\right)-U_{i}\left(s_{i},s_{-i}\right)]\;\in \;[-1,+1], \end{array}$$ (4)

*where A*_*i*_ = max_*s*_ *U*_*i*_(*s*) − min_*s*_ *U*_*i*_(*s*) > 0 *is the peak-to-peak amplitude of i’s payoffs*.

If $G_{N}$ is an *n* × 2 game and $S_{i}=\{\varphi_{i},\psi_{i}\}$, we have that *ℓ*_*i*_(*φ*_*i*_, *s*_−*i*_) = −*ℓ*_*i*_(*ψ*_*i*_, *s*_−*i*_); for the sake of simplicity, we write $\ell_{i}^{*}(s_{-i})=\ell_{i}(\varphi_{i},s_{-i})$ moving forward. Thus, the maximum number of unique values of $\ell_{i}^{*}(s_{j})$ per player in a binary normal-form game is 2^*n*−1^. For *n* = 2, we write

$$\begin{array}{c} \ell_{i}^{*}\left(s_{j}\right)=\frac{1}{A_{i}}[U_{i}\left(\varphi_{i},s_{j}\right)-U_{i}\left(\psi_{i},s_{j}\right)]\;\in \;[-1,+1], \end{array}$$ (5)

We now rewrite Eqs (2) and (3) in terms of Eqs (4) and (5) as

$$F_{i}\left(\varphi ,\psi \right)=\frac{1}{A_{i}}[U_{i}\left(\varphi_{i},\varphi_{j}\right)-U_{i}\left(\psi_{i},\varphi_{j}\right)]=-\ell_{i}\left(\psi_{i},\varphi_{j}\right)=\ell_{i}^{*}\left(\varphi_{j}\right),$$ (6)

and

$$G_{i}\left(\varphi ,\psi \right)=\frac{1}{A_{i}}[U_{i}\left(\varphi_{i},\psi_{j}\right)-U_{i}\left(\psi_{i},\psi_{j}\right)]=-\ell_{i}\left(\psi_{i},\psi_{j}\right)=\ell_{i}^{*}\left(\psi_{j}\right).$$ (7)

If the labels *φ* and *ψ* are swapped above, *F*_*i*_(*ψ*, *φ*) = −*G*_*i*_(*φ*, *ψ*) and *G*_*i*_(*ψ*, *φ*) = −*F*_*i*_(*φ*, *ψ*).

In binary games, equilibrium conditions depend only on the signa of each player’s loss of deviating away from a collective strategy (see Definitions A.2 and A.2a in the S1 Appendix). More precisely, $s^{⋆}=\langle s_{i}^{⋆},s_{-i}^{⋆}\rangle \in S$ is a PNE if

$$\underset{i\in N}{\text{min}}\ell_{i}\left(s_{i}^{⋆},s_{-i}^{⋆}\right)\geq 0.$$

In a 2 × 2 game, if both players’ structural fear or greed values fall in the same region (as is the case for symmetric games), then

$$\begin{array}{c} \text{sgn}\;\ell_{i}\left(s_{i},s_{j}\right)=\text{sgn}\;\ell_{j}\left(s_{j},s_{i}\right), \end{array}$$ (8)

for some $s_{i}\in S_{i}=\{\varphi_{i},\psi_{i}\}$ and some $s_{j}\in S_{j}=\{\varphi_{j},\psi_{j}\}$, where sgn:R↦{−1,0,+1} is the signum function. If both sides of Eq (8) are equal to +1, then *s* = 〈*s*_*i*_, *s*_*j*_〉 must be a strict PNE. We denote the set of PNE as $S^{⋆}$. We use the bimatrix notation in Eq (1) to compare cardinal payoffs with the signa of the rescaled deviation losses and evaluate $S^{⋆}$ in a 2 × 2 game:

$$\begin{array}{cc} {}[\left\langle \;U_{i},U_{j}\;\right\rangle {]}_{2\times 2} & ∼[\;\begin{array}{cc} \left\langle \;\text{sgn}\;\ell_{i}^{*}\left(\varphi_{j}\right),\;\text{sgn}\;\ell_{j}^{*}\left(\;\;\varphi_{i}\right)\;\right\rangle & \left\langle \;\text{sgn}\;\ell_{i}^{*}\left(\psi_{j}\right),-\text{sgn}\;\ell_{j}^{*}\left(\;\;\varphi_{i}\right)\;\right\rangle \\ \left\langle \;-\text{sgn}\;\ell_{i}^{*}\left(\varphi_{j}\right),\;\text{sgn}\;\ell_{j}^{*}\left(\psi_{i}\right)\;\right\rangle & \left\langle \;-\text{sgn}\;\ell_{i}^{*}\left(\psi_{j}\right),-\text{sgn}\;\ell_{j}^{*}\left(\psi_{i}\right)\;\right\rangle \end{array}\;]\\ & ∼[\;\begin{array}{cc} \left\langle \;\text{sgn}\;F_{i},\;\text{sgn}\;F_{j\;}\;\right\rangle & \left\langle \;\text{sgn}\;G_{i},-\text{sgn}\;F_{j\;}\;\right\rangle \\ \left\langle \;-\text{sgn}\;F_{i},\;\text{sgn}\;G_{j}\;\right\rangle & \left\langle \;-\text{sgn}\;G_{i},-\text{sgn}\;G_{j}\;\right\rangle \end{array}\;]. \end{array}$$ (9)

**Fig 3:**
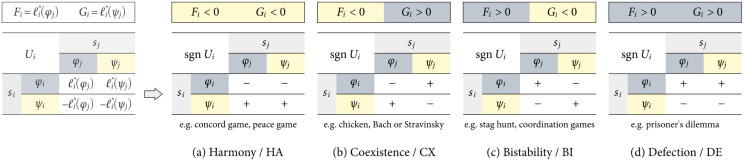
Classification of strategy dynamics in 2 × 2 social dilemma games in terms of the individual payoffs *U*_*i*_(*s*_*i*_, *s*_*j*_) and their relative differences (+ versus −). An economically rational player would presumably prefer those collective strategies marked with a plus sign (+) over those marked with a minus sign (−). In the case of indifference dynamics, notice that both *s*_*i*_ rows are equal, implying that player *i*’s payoff only depends on player *j*’s selection.

Mapping a 2 × 2 game to the bimatrix form in Eq (9) allows us to identify any PNE in it more quickly and how they relate to the structural fear and greed factors. Fig 3 reintroduces the main 2 × 2 strategy dynamical domains using the aforementioned notation. As an example, the payoffs in a symmetric game with harmony dynamics can be written as

$$\begin{array}{c} {}[\left\langle \;U_{i},U_{j}\;\right\rangle {]}_{2\times 2}=[\;\begin{array}{cc} \left\langle 1,1\right\rangle & \left\langle 3,2\right\rangle \\ \left\langle 2,3\right\rangle & \underline{\left\langle 4,4\right\rangle } \end{array}\;]∼[\;\begin{array}{cc} \left\langle -,-\right\rangle & \left\langle -,+\right\rangle \\ \left\langle +,-\right\rangle & \underline{\left\langle +,+\right\rangle } \end{array}\;]; \end{array}$$

we underline the cells where each of the two signa are greater than or equal to zero. Thus in the game above, there is one PNE, *ψ*, since *F*_*i*_, *F*_*j*_ < 0 and *G*_*i*_, *G*_*j*_ < 0, and

$$\text{sgn}\;\ell_{i}\left(\varphi_{i},\;\cdot \;\right)=-1=\text{sgn}\;\ell_{j}\left(\varphi_{j},\;\cdot \;\right),$$

implies that both players would always prefer *ψ*_*i*_ (second row) and *ψ*_*j*_ (second column) over *φ*_*i*_ and *φ*_*j*_. Similarly, in a symmetric game with coexistence dynamics, for example

$$\begin{array}{c} {}[\left\langle \;U_{i},U_{j}\;\right\rangle {]}_{2\times 2}=[\;\begin{array}{cc} \left\langle 1,1\right\rangle & \underline{\left\langle 4,2\right\rangle }\\ \underline{\left\langle 2,4\right\rangle } & \left\langle 3,3\right\rangle \end{array}\;]∼[\;\begin{array}{cc} \left\langle -,-\right\rangle & \underline{\left\langle +,+\right\rangle }\\ \underline{\left\langle +,+\right\rangle } & \left\langle -,-\right\rangle \end{array}\;], \end{array}$$

there are two PNE, $S^{⋆}$ = {〈*φ*_*i*_, *ψ*_*j*_〉, 〈*ψ*_*i*_, *φ*_*j*_〉}, the off-diagonal strategies, since *F*_*i*_ < 0 and *G*_*j*_ > 0—likewise, *F*_*j*_ < 0 and *G*_*i*_ > 0—imply

$$\begin{array}{cc} -\text{sgn}\;F_{i} & =+1=\text{sgn}\;G_{j}\\ -\text{sgn}\;\ell_{i}^{*}\left(\varphi_{j}\right) & =+1=\text{sgn}\;\ell_{j}^{*}\left(\psi_{i}\right)\\ \text{sgn}\;\ell_{i}\left(\psi_{i},\varphi_{j}\right) & =+1=\text{sgn}\;\ell_{j}\left(\varphi_{j},\psi_{i}\right), \end{array}$$

indicating that not taking the same action would be the most economically rational outcome.

A game with one or more PNE can also be formed by two individual payoff matrices exhibiting different strategy dynamics. We provide two examples. One: we can model *U*_*i*_ after the harmony payoffs in Fig 3(a) and *U*_*j*_ after the (transposed) defection payoffs in Fig 3(d); using the bimatrix game notation in Eq (9), we can write

$$\begin{array}{c} {}[\left\langle \;U_{i},U_{j}\;\right\rangle {]}_{2\times 2}=[\;\begin{array}{cc} \left\langle 0,0\right\rangle & \left\langle 0,-1\right\rangle \\ \underline{\left\langle 1,0\right\rangle } & \left\langle 1,-1\right\rangle \end{array}\;]∼[\;\begin{array}{cc} \left\langle -,+\right\rangle & \left\langle -,-\right\rangle \\ \underline{\left\langle +,+\right\rangle } & \left\langle +,-\right\rangle \end{array}\;], \end{array}$$ (10)

where the collective strategy 〈*ψ*_*i*_, *φ*_*j*_〉 is the only equilibrium in this game. The individual strategies in Eq (10) are also *perfectly limited*, meaning they yield an individual payoff that depends only on each player’s strategy [35]. Using Eqs (6) and (7), we calculate the structural fear and greed for players *i* and *j* as *F*_*i*_ = *G*_*i*_ = −1 < 0 (both factors support *ψ*_*i*_) and *F*_*j*_ = *G*_*j*_ = +1 > 0 (both factors support *φ*_*j*_), respectively. Two: let us reuse the coexistence payoffs to model *U*_*i*_ and (transposed) bistability payoffs per Fig 3(c) to model *U*_*j*_ and write

$$\begin{array}{c} {}[\left\langle \;U_{i},U_{j}\;\right\rangle {]}_{2\times 2}=[\;\begin{array}{cc} \left\langle 0,0\right\rangle & \left\langle 0,-1\right\rangle \\ \left\langle 1,0\right\rangle & \left\langle -1,1\right\rangle \end{array}\;]∼[\;\begin{array}{cc} \left\langle -,+\right\rangle & \left\langle +,-\right\rangle \\ \left\langle +,-\right\rangle & \left\langle -,+\right\rangle \end{array}\;]; \end{array}$$ (11)

this time, however, there are no PNE. Also, each player only has one perfectly limited strategy: *φ*_*i*_ and *φ*_*j*_ always yield a zero payoff of zero to players *i* and *j* regardless of the other’s choice of individual strategy. The values of structural fear and greed for players *i* and *j* are 〈*F*_*i*_, *G*_*i*_〉 = 〈−1/2, +1/2〉 and 〈*F*_*j*_, *G*_*j*_〉 = 〈+1/2, −1/2〉, respectively. If only the signa of each player’s structural fear and greed factors are considered, the bimatrix game in Eq (11) is qualitatively equivalent to the 2 × 2 zero-sum game

$$\begin{array}{c} {}[\left\langle \;U_{i},U_{j}\;\right\rangle {]}_{2\times 2}=[\;\begin{array}{cc} \left\langle -1,1\right\rangle & \left\langle 1,-1\right\rangle \\ \left\langle 1,-1\right\rangle & \left\langle -1,1\right\rangle \end{array}\;]. \end{array}$$ (12)

In the following section, we extend the definitions of $\ell_{i}^{*}$, *F*_*i*_, and *G*_*i*_ to normal-form games with any number of players *n* ≥ 2 and binary strategy sets $S_{i}=\{\varphi_{i},\psi_{i}\}$ for every i∈N.

## 3 Strategy dynamics in *n* × 2 games

In this section, we reformulate the rescaled deviation losses $\ell_{i}^{*}(s_{-i})=\ell_{i}(\varphi_{i},s_{-i})$ for a player i∈N in an *n* × 2 game in terms of the possible pure individual strategies played by subsets of N\{i}. We focus on the scenarios in which standpoint player *i* breaks their game into smaller 2 × 2 games by making two types of conjectures about the possible individual actions of others: 1) some players will coordinate their strategies and act as one, and 2) some other players have already made their choice of individual strategy and will not change their minds. Later, we apply the proposed framework to describe individual and collective strategy dynamics in 3 × 2 games and characterize social dilemmas with three players.

### 3.1 Dissecting fear and greed in *n* × 2 games: Strategic hindrance

We start by defining *player-reduced binary normal-form games*:

**Definition 2** *Let* $G_{N}=(N,{\left(S_{i}\right)}_{i\in N},{\left(U_{i}\right)}_{i\in N})$ *be a normal-form game; let* i∈N *be a standpoint player in game* $G_{N}$ ; *let* K⊂N\{i} *be a proper subset of players in* N *excluding i; and let* J=N\(K∪{i}) *be the non-empty set of players different from i that are not in* K. *Assume, without loss of generality, that player i anticipates a collective action* $s_{K}$ *by the players in* K. *From player i’s standpoint, the normal-form game* $G_{N}$ *reduces to*

$$\begin{array}{c} G_{N\;\backslash \;K}=(\;N\;\backslash \;K,(S_{i}{)}_{i\in N\;\backslash \;K},(U_{i}\left(\;\cdot \;,s_{K}\right){)}_{i\in N\;\backslash \;K}\;). \end{array}$$ (13)

*That is*, $G_{N\;\backslash \;K}$ *is a player-reduced game observed by player i within* $G_{N}$.

We use Definition 2 to reformulate Eq (4) in terms of *s*_*i*_ ∈ {*φ*_*i*_, *ψ*_*i*_} and $s_{-i}=\langle s_{\;J},\;s_{K}\rangle$ as

$$\begin{array}{c} \ell_{i}^{*}\left(s_{\;J},\;s_{K}\right)=A_{i}^{-1}\cdot [U_{i}\left(\varphi_{i},\;s_{\;J},\;s_{K}\right)-U_{i}\left(\psi_{i},\;s_{\;J},\;s_{K}\right)]\;\in \;[-1,+1]; \end{array}$$ (14)

where $s_{\;J}\in S_{i}^{\left|J\right|}$, |J|∈[1‥n−1]; and $s_{K}\in S_{i}^{\left|K\right|}$, |K|∈[0‥n−2].

Next, we characterize player *i*’s fear and greed in game $G_{N\;\backslash \;K}$ assuming a coordinated action $s_{\;J}\in \{\varphi_{\;J},\psi_{\;J}\}$ by player-set J and an unmovable action $s_{K}=\sigma_{K}$ by player-set J:

**Definition 3** *Let* $G_{N\;\backslash \;K}$ *be a player-reduced game observed by player i, where* $s_{K}=\sigma_{K}$ *is the collective strategy of the (excluded) players in* K . *Assume that, in the face of uncertainty, player i conjectures that all players in* J *act as one player with individual strategy* $s_{\;J}\in \{\varphi_{\;J},\psi_{\;J}\}$; *and that the players in* K *have adopted a presumably immovable collective strategy* $s_{K}=\sigma_{K}$ *of which player i is “certain.” The values of structural fear and greed specific to player i concerning a unanimous course of action by the players in* J *are*

$$F_{i}^{\left(\sigma_{K}\right)}\left(\varphi ,\psi \right)=\ell_{i}^{*}\left(\varphi_{\;J},\sigma_{K}\right)=A_{i}^{-1}\cdot [U_{i}\left(\varphi_{i},\varphi_{J\;},\sigma_{K}\right)-U_{i}\left(\psi_{i},\varphi_{\;J},\sigma_{K}\right)]$$ (15)

*and*

$$G_{i}^{\left(\sigma_{K}\right)}\left(\varphi ,\psi \right)=\ell_{i}^{*}\left(\psi_{\;J},\sigma_{K}\right)=A_{i}^{-1}\cdot [U_{i}\left(\varphi_{i},\psi_{\;J},\sigma_{K}\right)-U_{i}\left(\psi_{i},\psi_{\;J},\sigma_{K}\right)],$$ (16)

*where A*_*i*_ = max_*s*_ *U*_*i*_(*s*) − min_*s*_ *U*_*i*_(*s*) > 0 *and* $s_{K}\in S_{i}^{\left|K\right|}$.

A few of notes on Definition 3:

- Setting N={i,j}—which implies J={j} and K=∅—turns Eqs (15) and (16) into Eqs (6) and (7), respectively.
- We intend the assumption of a fixed $s_{K}=\sigma_{K}$ to represent the scenarios in which each player k∈K are committed to playing a particular individual strategy $\sigma_{k}\in S_{k}$ and player *i* both know of their intentions and expects them to be fully realized; we call this a *type I player-reduced binary game: stationary* K-*players*.
- A second type of player-reduced binary game concerns the scenario in which one or more players in K are not expected to commit to $\sigma_{K}$; we call this a *type II player-reduced binary game: reversing* K-*players* (see Section A.2 in the S1 Appendix for more details).

We now define the *strategic hindrance* of standpoint player *i* as the space of structural fear and greed values for all possible type I player-reduced games where no more than *n* − 2 players are fixed.

**Definition 4** *Given a binary n-player normal-form game* $G_{N}$ *with individual strategy set* $S_{i}=\{\varphi_{i},\psi_{i}\}$ , *we define the space of structural fear and greed values, or strategic hindrance, of a player* i∈N *as*

$$\begin{array}{c} {\text{H}}_{i}^{\left(n\right)}\left(\varphi ,\psi \right)=[\begin{array}{c} F_{i}^{\left(\sigma_{K}\right)}\left(\varphi ,\psi \right)\\ G_{i}^{\left(\sigma_{K}\right)}\left(\varphi ,\psi \right) \end{array}{]}_{\times g\left(n\right)}^{\text{T}}\;\;=\;\;[\begin{array}{c} \ell_{i}^{*}\left(\varphi_{\;J},\sigma_{K}\right)\\ \ell_{i}^{*}\left(\psi_{\;J},\sigma_{K}\right) \end{array}{]}_{\times g\left(n\right)}^{\text{T}}\;\;,\;\;\forall J\;\cup K=N\;\backslash \;\left\{i\right\},\;J\;\neq \emptyset . \end{array}$$ (17)

*where g(n) is the number of possible player-reduced binary games* $G_{N\;\backslash \;K}$ *with stationary* K-*players (type I)*.

The total number of possible type I player-reduced binary games with fixed $s_{K}=\sigma_{K}$ is (see Section A.2 in the S1 Appendix):

$$\begin{array}{c} g\left(n\right)=3^{n-1}-2^{n-1},\;\;\;\forall \;n\geq 1. \end{array}$$ (18)

There are only 2^*n*−1^ possible $\ell_{i}^{*}(s_{-i})$ that can be input as one of the 2 ⋅ *g*(*n*) entries of the strategic hindrance space in Eq (4). For instance, if *n* = 3, *g*(*n*) = 5, and each of the 2 × 5 = 10 entries in player *i*’s strategic hindrance space takes one of the 2^3−1^ = 4 values of $\ell_{i}^{*}(s_{-i})$. This implies that some deviation losses appear more than once and thus impact the emergent strategy dynamics more than others. We quantify such an impact using the function

$$\begin{array}{c} \gamma \left(n_{\varphi \backslash i},\;n_{\psi \backslash i}\right)=2^{n_{\varphi \backslash i}}+2^{n_{\psi \backslash i}}-2, \end{array}$$ (19)

which counts how many entries of each player’s strategic hindrance space are equal to $\ell_{i}^{*}(s_{-i})$ based solely on the number of players in N\{i} that play either *φ*_*j*_ and *ψ*_*j*_, *n*_*φ*\*i*_ and *n*_*ψ*\*i*_, satisfying *n*_*φ*\*i*_ + *n*_*ψ*\*i*_ = *n* − 1. The value of *γ* in Eq (19) is associated to the number of type I player-reduced binary games in Eq (18) via the following alternative formula for *g*(*n*) (see Section A.3 in the S1 Appendix):

$$\begin{array}{c} g\left(n\right)=\frac{1}{2}[\sum_{k=0}^{n-1}\gamma \left(k,\;n-1-k\right)\cdot C\left(n-1,\;k\right)]; \end{array}$$ (20)

where $C(n-1,k)=\left(\begin{array}{c} n-1\\ k \end{array}\right)$ is the binomial coefficient and *k* counts the entries in *s*_−1_ that are equal to either *φ*_*j*_ or *ψ*_*j*_—note that Eqs (19) and (20) are symmetric with respect to strategy, that is *γ*(*n*_*φ*\*i*_, *n*_*ψ*\*i*_) = *γ*(*n*_*ψ*\*i*_, *n*_*φ*\*i*_). For instance, if *n* = 3 and N={1,2,3}, the values of *γ* associated with each possible *s*_−1_ are

$$\begin{array}{c} \begin{array}{ccccc} & \text{for}\; & s_{-1}=\left\langle \varphi_{2},\varphi_{3}\right\rangle ,\; & n_{\varphi \backslash i}=2\;\text{and}\;n_{\psi \backslash i}=0 & \;\Rightarrow \;\gamma (2,\;0)=3;\\ & \text{for}\; & s_{-1}=\left\langle \varphi_{2},\psi_{3}\right\rangle ,\; & n_{\varphi \backslash i}=1\;\text{and}\;n_{\psi \backslash i}=1 & \;\Rightarrow \;\gamma (1,\;1)=2;\\ & \text{for}\; & s_{-1}=\left\langle \psi_{2},\varphi_{3}\right\rangle ,\; & n_{\varphi \backslash i}=1\;\text{and}\;n_{\psi \backslash i}=1 & \;\Rightarrow \;\gamma (1,\;1)=2;\\ \text{and} & \text{for}\; & s_{-1}=\left\langle \psi_{2},\psi_{3}\right\rangle ,\; & n_{\varphi \backslash i}=0\;\text{and}\;n_{\psi \backslash i}=2 & \;\Rightarrow \;\gamma (0,\;2)=3; \end{array} \end{array}$$

and, per Eq (20), *g*(3) = (1/2) ⋅ [*γ*(0, 2) ⋅ (1) + *γ*(1, 1) ⋅ (2) + *γ*(2, 0) ⋅ (1)] = (1/2) ⋅ 10 = 5.

### 3.2 Strategic hindrance in 3 × 2 games

This section inspects the information that can be extracted from a strategic hindrance of a player in a 3 × 2 game when represented in a Euclidean plane. Solving Eq (17) for *n* = 3, player *i*’s strategic hindrance can be expressed as:

$$\begin{array}{c} {\text{H}}_{i}^{\left(3\right)}\left(\varphi ,\psi \right)=\frac{1}{A_{i}}{\left[\begin{array}{ccccc} \ell_{i}^{*}\left(\varphi_{j},\;\varphi_{k}\right) & \;\;\;\;\ell_{i}^{*}\left(\varphi_{j},\;\psi_{k}\right) & \;\;\;\;\ell_{i}^{*}\left(\varphi_{j},\;\varphi_{k}\right) & \;\;\;\;\ell_{i}^{*}\left(\psi_{j},\;\varphi_{k}\right) & \;\;\;\;\ell_{i}^{*}\left(\varphi_{j},\;\varphi_{k}\right)\\ \ell_{i}^{*}\left(\psi_{j},\;\varphi_{k}\right) & \;\;\;\;\ell_{i}^{*}\left(\psi_{j},\;\psi_{k}\right) & \;\;\;\;\ell_{i}^{*}\left(\varphi_{j},\;\psi_{k}\right) & \;\;\;\;\ell_{i}^{*}\left(\psi_{j},\;\psi_{k}\right) & \;\;\;\;\ell_{i}^{*}\left(\psi_{j},\;\psi_{k}\right) \end{array}\right]}^{\text{T}}. \end{array}$$ (21)

**Fig 4:**
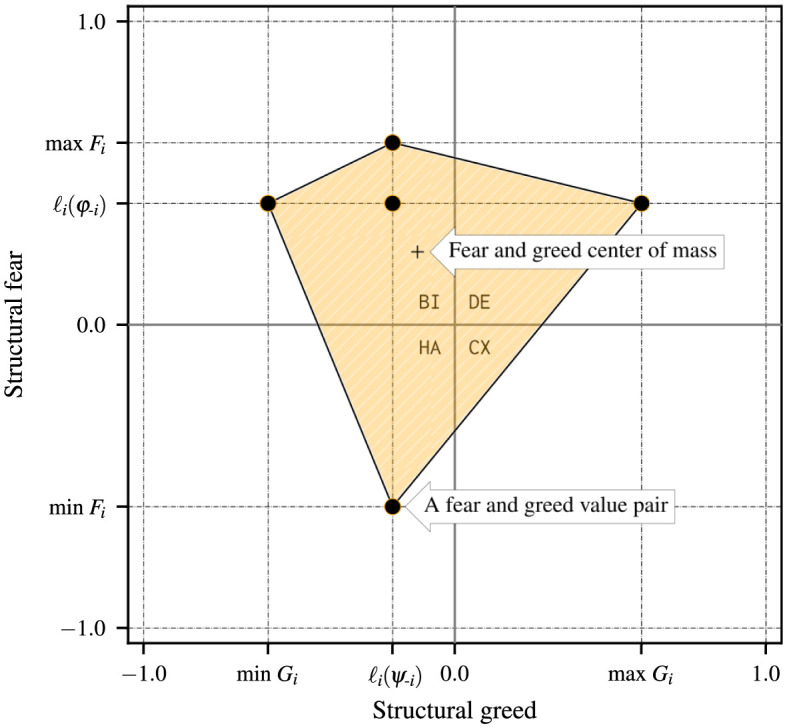
Sample strategic hindrance space ${\text{H}}_{i}^{\left(3\right)}(\varphi ,\psi )$ in a 3 × 2 game with *g*(3) = 5 unique type I player-reduced games observed by standpoint player *i*. The payoff structure is shown in Fig 5.

Table 1 lists each of the corresponding *g*(3) = 5 structural fear and greed value pairs for *n* = 3 in terms of the rescaled deviation losses $\ell_{i}^{*}(s_{j},s_{k})$. We use rectangular coordinates to describe a generic individual strategic hindrance space in Fig 4. In Fig 6, we add strategic hindrance spaces for the remaining two players and discuss how the visualization is useful for understanding the stability of collective actions in the game. We repeat this assessment after modifying the payoffs of one of the players, namely by multiplying by −1, which results in the strategic hindrance spaces in Fig 9.

**Table 1:** Strategic hindrance space ${\text{H}}_{i}^{\left(3\right)}(\varphi ,\psi )$ for player *i* in a 3 × 2 game.

			${\text{H}}_{i}^{\left(3\right)}(\varphi ,\psi )$					
J	K	$\sigma_{K}$	$F_{i}^{\left(\sigma_{K}\right)}$			$G_{i}^{\left(\sigma_{K}\right)}$		
{*j*}	{*k*}	*φ*_*k*_	$\ell_{i}^{*}(\varphi_{j},\varphi_{k})$					$\ell_{i}^{*}(\psi_{j},\varphi_{k})$
		*ψ*_*k*_		$\ell_{i}^{*}(\varphi_{j},\psi_{k})$		$\ell_{i}^{*}(\psi_{j},\psi_{k})$		
{*k*}	{*j*}	*φ*_*j*_	$\ell_{i}^{*}(\varphi_{j},\varphi_{k})$				$\ell_{i}^{*}(\varphi_{j},\psi_{k})$	
		*ψ*_*j*_			$\ell_{i}^{*}(\psi_{j},\varphi_{k})$	$\ell_{i}^{*}(\psi_{j},\psi_{k})$		
{*j*, *k*}	∅	n/a	$\ell_{i}^{*}(\varphi_{j},\varphi_{k})$			$\ell_{i}^{*}(\psi_{j},\psi_{k})$		

#### Generic individual 3 × 2 strategic hindrance space

A player’s strategic hindrance highlights the multiple modes of biases that are baked into the payoff structure. In a one-shot game, a player may rely on simple arithmetic to play the pure strategy that maximizes their expected value. This may be the case in games with more regular payoff structures that produce fewer unique fear and greed value pairs per player, usually falling entirely within the same strategy dynamical domain—e.g., the social dilemmas analyzed in Section A.4 in S1 Appendix. All structural fear and greed value pairs that form a strategic hindrance space in a 3 × 2 game can be contained within a convex polygon with at most four sides—we conjecture that the number of sides of the convex polygon containing all fear and greed value pairs in an individual strategic hindrance space is at most 4(*n* − 2) for *n* > 2.

**Fig 5:**
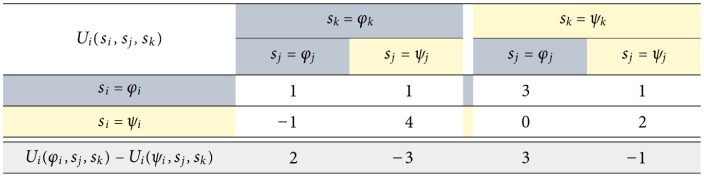
Sample individual payoffs and absolute deviation losses in a 3 × 2 game. The resulting strategic hindrance space is shown in Fig 4; the peak-to-peak payoff amplitude is *A*_*i*_ = 5.

The payoffs and absolute deviation losses that produce the strategic hindrance space in Fig 4 are provided in Fig 5. Using Eqs (14) and (15)–(17) we obtain player *i*’s strategic hindrance space:

$$\begin{array}{c} {\text{H}}_{i}^{\left(3\right)}\left(\varphi ,\psi \right)=[\begin{array}{c} F_{i}^{\left(\sigma_{K}\right)}\\ G_{i}^{\left(\sigma_{K}\right)} \end{array}{]}_{\times 5}^{\text{T}}\;\;=\frac{1}{5}{\left[\begin{array}{ccccc} +2 & +3 & +2 & -3 & +2\\ -3 & -1 & +3 & -1 & -1 \end{array}\right]}^{\text{T}}. \end{array}$$

The five fear and greed value pairs in the above strategic hindrance space are all unique and fall across three 2 × 2 strategy dynamical domains—harmony (1), defection (1), and bistability (3). The center of mass is located at the average structural fear and greed coordinates, $\bar{F_{i}}=6/25$ and $\bar{G_{i}}=-3/25$. There are always *γ*(*n* − 1, 0) pairs along the ordinate $\ell_{i}^{*}(\varphi_{-i})$ and *γ*(0, *n* − 1) along the abscissa $\ell_{i}^{*}(\psi_{-i})$, with their intersection representing the player-reduced binary game where all other players play as one.

In games where players’ incentives are sparser, pre-existing beliefs of what others would do could make a player dismiss the likelihood of specific outcomes. The strategic hindrance space represents the spectrum of such beliefs, each of which a standpoint player would weigh according to a perceived chance, guiding their presumptions about the course of the game. If the standpoint player in our hypothetical 3 × 2 game example were to assign the same weight to all instances in Fig 4, the most prevalent strategy dynamics affecting their decision-making process would be structural bistability. This would involve the anticipation of aligning their strategies with at least one of the two other players. Since the remaining emergent strategy dynamics are harmony and defection, the standpoint player would prefer everyone to pursue a unanimous course of action, fully embracing either *ψ* or *φ*.

#### Inferring collective stability from multiple strategic hindrance spaces

**Fig 6:**
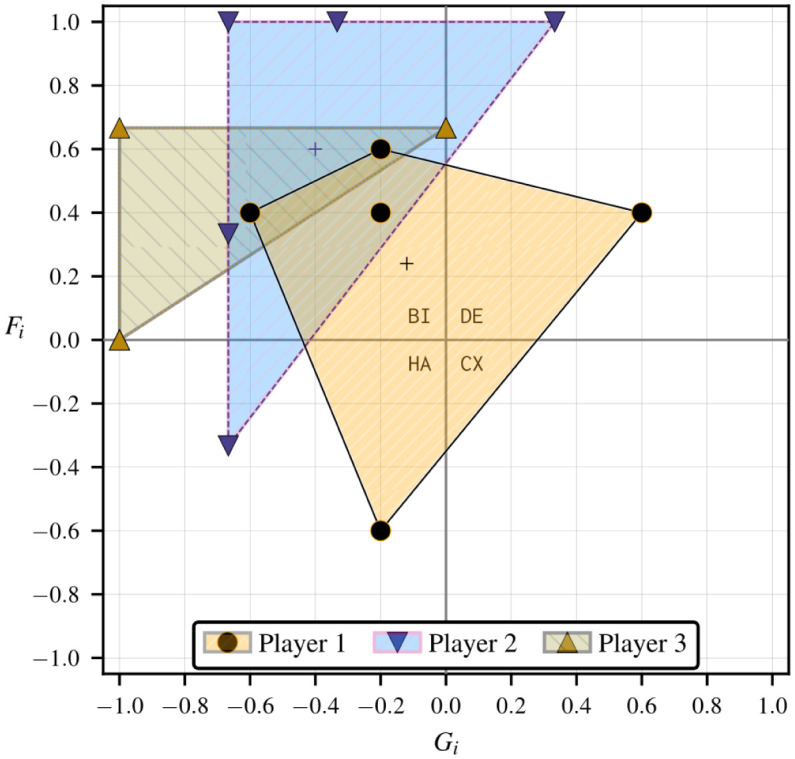
Strategic hindrance spaces in the sample 3 × 2 game in Fig 7. Player 1’s payoff structure is taken from Fig 5, so their strategic hindrance space is the same as in Fig 4. Bistability dynamics prevail across the strategic hindrance spaces of all players, while the emergence of harmony and defection may add further appeal to the diagonal collective strategies *ψ* and *φ*.

**Fig 7:**
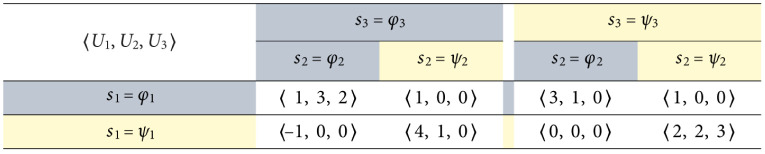
Sample 3 × 2 game used to generate the strategic hindrance spaces in Fig 6. Player 1’s incentives are modeled after the payoff structure in Fig 5. This game has two PNE: *φ* and *ψ*.

The strategic hindrance space in Fig 4 is a prognostic of a single player’s stability of rational strategic action based on their payoff structure. Suppose all other players shared similar payoff structures; in this scenario, we can anticipate the emergence of PNE coherent with the individual rational preferences coinciding in the same strategy dynamical domains. In some circumstances, we can also repeal PNE by inverting some players’ strategy dynamics, breaking compatibilities apart. For the grand game to exhibit PNE consistent with player 1’s preferences, the payoff structures of the other players must also result in a combination of harmony, bistability, and defection dynamics. Fig 6 highlights such a scenario with similar strategic hindrances, with player 2’s strategic hindrance space spanning the same three strategy dynamical domains as player 1’s, and all player 3’s fear and greed value pairs (weakly) falling under bistability. The baseline 3 × 2 game is presented in Fig 7. The PNE set in this game, $S^{⋆}=\{\varphi ,\psi \}$, results from the intersection between the rational preferences of all three players under bistability dynamics (which already favor *φ* and *ψ*) and the strict stability under harmony and defection dynamics for players 1 and 2.

**Fig 8:**
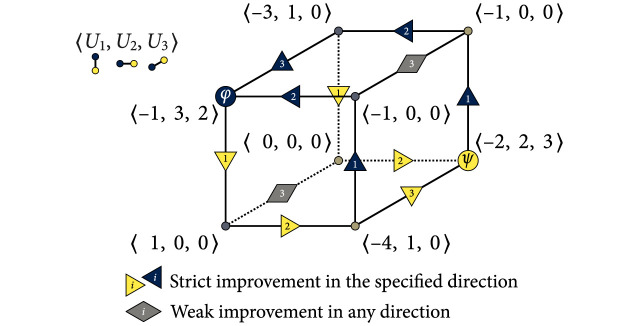
Modified 3 × 2 game after negating player 1’s payoffs in Fig 7, resulting in no PNE. This 3-cube representation highlights the anticipated lack of convergence toward a collective strategy.

**Fig 9:**
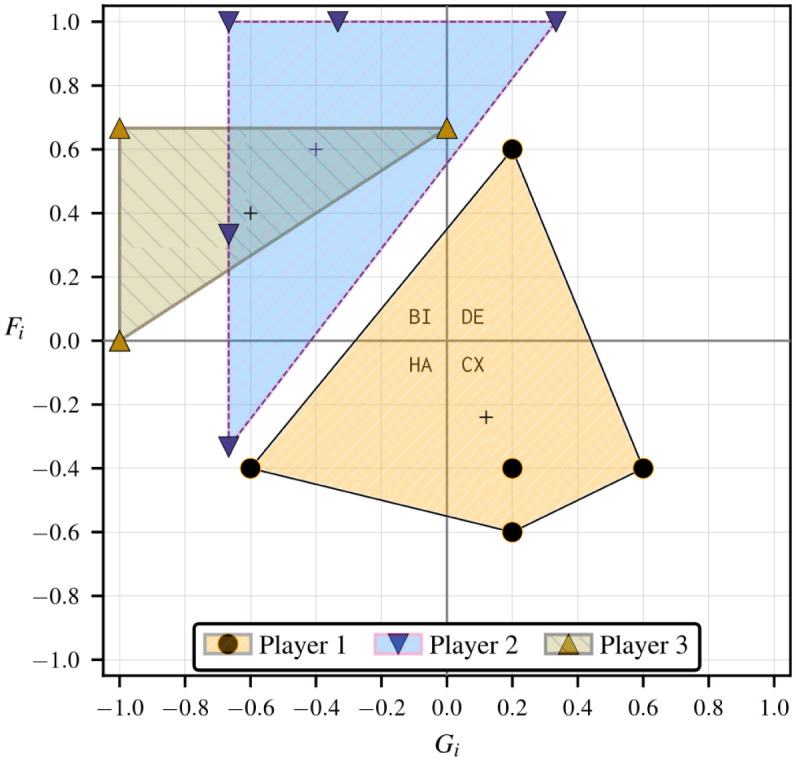
Strategic hindrance spaces in the 3 × 2 game in Fig 8. All strategic hindrance spaces but player 1’s are the same as in Fig 6—the payoff structures of players 2 and 3 are taken from Fig 7. The coexistence dynamics that emerge for player 1 indicate a greater incentive for refusing to align with the strategies of players 2 and 3.

In contrast with the strategic hindrance spaces in Fig 6, we can create a scenario where there is no set of type I player-reduced game observed by all players that strictly falls under the same 2 × 2 strategy dynamical domain. We can achieve this by negating player 1’s payoffs in Fig 7. The resulting game is shared in Fig 8 using an alternative 3-cub representation to highlight the directions of strict and weak payoff improvement to identify PNE, with the vertical, horizontal, and depth axes listing the individual strategies for players 1, 2, and 3, respectively. Each vertex represents a collective strategy outcome. The direction of each orthogonal edge connecting two vertices is determined by the sign of the individual deviation loss associated with it. A vertex is a PNE if it is a sink [36], meaning that all arcs connected to it are incoming [36]. There are no sinks in Fig 6; so, there are no PNE. The deviation losses and strategic hindrance spaces in this game are provided in Table 2 and visualized in Fig 9.

**Table 2:** Deviation losses and strategic hindrance spaces for the 3 × 2 game in Fig 8. All spaces are visualized in Fig 9. The results for players 2 and 3 are also shared in Fig 6.

	Player 1	Player 2	Player 3
$\ell_{i}^{*}$	$\ell_{1}^{*}(\varphi_{2},\varphi_{3})=\frac{1}{5}(-1-1)=-\frac{2}{5}$	$\ell_{2}^{*}(\varphi_{3},\varphi_{1})=\frac{1}{3}(3-0)=+\;1$	$\ell_{3}^{*}(\varphi_{1},\varphi_{2})=\frac{1}{3}(2-0)=+\frac{2}{3}$
	$\ell_{1}^{*}(\varphi_{2},\;\psi_{3})=\frac{1}{5}(-3-0)=-\frac{3}{5}$	$\ell_{2}^{*}(\varphi_{3},\;\psi_{1})=\frac{1}{3}(1-0)=+\frac{1}{3}$	$\ell_{3}^{*}(\varphi_{1},\psi_{2})=\frac{1}{3}(0-0)=0$
	$\ell_{1}^{*}(\;\psi_{2},\varphi_{3})=\frac{1}{5}(-1+4)=+\frac{3}{5}$	$\ell_{2}^{*}(\;\psi_{3},\varphi_{1})=\frac{1}{3}(0-1)=-\frac{1}{3}$	$\ell_{3}^{*}(\psi_{1},\varphi_{2})=\frac{1}{3}(0-0)=0$
	$\ell_{1}^{*}(\;\psi_{2},\;\psi_{3})=\frac{1}{5}(-1+2)=+\frac{1}{5}$	$\ell_{2}^{*}(\;\psi_{3},\;\psi_{1})=\frac{1}{3}(0-2)=-\frac{2}{3}$	$\ell_{3}^{*}(\;\psi_{1},\;\psi_{2})=\frac{1}{3}(0-3)=-\;1$
${\text{H}}_{i}^{\left(3\right)}$	$\frac{1}{5}\left[\begin{array}{ccccc} -2 & -3 & -2 & +3 & -2\\ +3 & +1 & -3 & +1 & +1 \end{array}\right]$	$\frac{1}{3}\left[\begin{array}{ccccc} +3 & +1 & +3 & -1 & +3\\ -1 & -2 & +1 & -2 & -2 \end{array}\right]$	$\frac{1}{3}[\begin{array}{ccccc} 0 & -3 & 0 & -3 & -3\\ +2 & 0 & +2 & 0 & +2 \end{array}]$

Negating an individual payoff structure causes observed harmony dynamics to turn into defection; it also causes bistability to turn into coexistence (and vice-versa). Harmony and defection are both dominance dynamics and thus are compatible, as described for 2 × 2 games in Section 2.3 and Eq (10)—in Section A.4 in S1 Appendix, we discuss *public goods games*, which have a single PNE and where the aspiration level of each player can be set to result in either pure defection or pure harmony dynamics. The same cannot be said about coexistence and bistability. In the 2 × 2 games in Eqs (11) and (12), we observe that combining positive and negative frequency dependence may result in the absence of PNE. Coexistence dynamics do not favor a unanimous course of action. Player 1’s coexistence-based preferences in Fig 9 cancel out the intersecting harmony-bistability-defection preferences of players 2 and 3 that favor *φ* and *ψ*.

**Fig 10:**
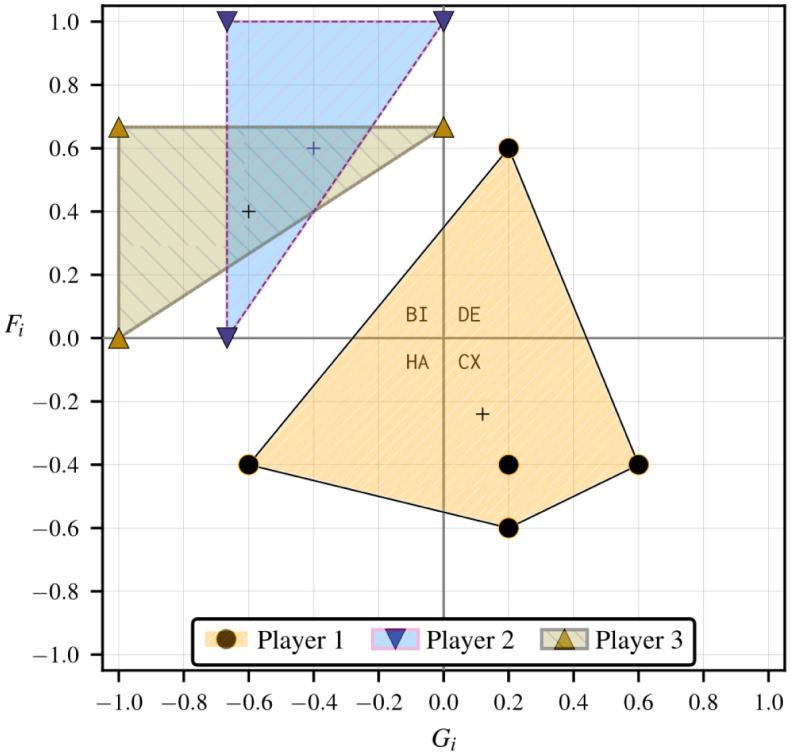
Altered strategy dynamics derived from the 3 × 2 game in Fig 8 after making *U*_2_(*s*) = 0 at *s* = 〈*φ*_1_, *φ*_2_, *ψ*_3_〉 and *s* = 〈*ψ*_1_, *ψ*_2_, *φ*_3_〉, resulting in $\ell_{2}^{*}(\;\varphi_{3},\psi_{1})=\ell_{2}^{*}(\;\psi_{3},\varphi_{1})=0$ in Table 2. The strategic hindrance spaces of players 2 and 3 are akin to those in a unanimity game (Fig A.5 in S1 Appendix) characterized in Section A.4 in S1 Appendix. The PNE set is $S^{⋆}=\{\langle \varphi_{1},\psi_{2},\psi_{3}\rangle ,\;\langle \psi_{1},\varphi_{2},\varphi_{3}\rangle \}$.

It is still possible to create a 3 × 2 game where the incidence of coexistence and bistability results in the emergence of PNE. This can be done by mitigating harmony and defection. For instance, in Fig 8, making *U*_2_(*s*) = 0 at *s* = 〈*φ*_1_, *φ*_2_, *ψ*_3_〉 and *s* = 〈*ψ*_1_, *ψ*_2_, *φ*_3_〉 makes the rescaled deviation losses $\ell_{2}^{*}(\;\varphi_{3},\psi_{1})$ and $\ell_{2}^{*}(\;\psi_{3},\varphi_{1})$ in Table 2 become zero, turning all but one of the *g*(3) = 5 type I player-reduced games into borderline bistability games with either *F*_2_ = 0 or *G*_2_ = 0. Player 2’s modified strategic hindrance is similar to that of player 3, as shown in Fig 10—*unanimity* games, in the following section, are characterized by the emergence of analogous borderline bistability dynamics for all players. The lower left and upper right vertices in Fig 8—respectively {〈*φ*_1_, *ψ*_2_, *ψ*_3_〉 and 〈*ψ*_1_, *φ*_2_, *φ*_3_〉}—weakly become sinks. These weak PNE result from the intersection of player 1’s coexistence-driven disfavoring of unanimity and players 2 and 3’s bistability-driven inclination to coordinate with other players. Here the incidence of player 1’s observed harmony and defection dynamics is negligible.

### 3.3 Application case: Integrability of an urban transit system

Here, we analyze the emergence of strategy dynamics in the strategic design for the integrability of a fictional urban transit system, highlighting the complexities that arise as the number of key design actors increases. In this example, we explore the concept of directional integrability [14], briefly discussed in Section 1.1, to assess how well the system-of-systems aligns with shared goals, considering the levels of managerial control and collaboration among its components.

#### The baseline 2 × 2 scenario

Let us consider two managerially and operationally independent system design actors:

- Irene, a software developer who was contracted by the public transportation authority to implement a passenger information system; and
- Jamie, an infrastructure contractor whom the regional transportation policy agency tasked to design and maintain the traffic management system.

Irene’s primary role is to help commuters plan their routes and access real-time information on buses, trams, and subways, while Jamie’s contribution to developing and managing road infrastructure and traffic critically impacts the flow of vehicles and the safety of passengers.

Each actor can independently choose an open or closed system strategy:

- *φ*_*i*_—closed system to leverage partial integrability: the system will take advantage of the capabilities of other open systems, but the latter cannot exploit the former.
- *ψ*_*i*_—open system to pursue synergistic integrability: the system’s capabilities can be fully leveraged by everyone.

**Fig 11:**
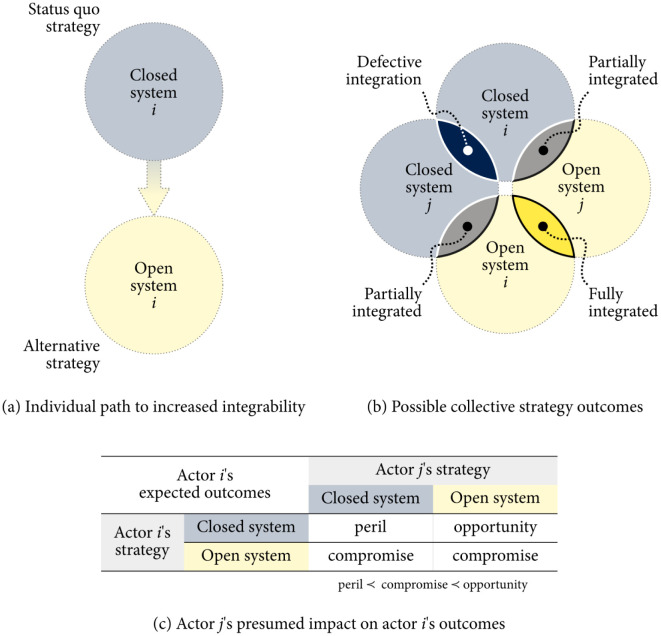
Increasing integrability strategically: (a) Each actor (say, actor *i*) decides whether to keep their system closed (status quo) or to allocate resources to making their system fully integrable for the benefit of other systems (alternative strategy); (b) The expected outcomes resulting from the interaction between two systems, *i* and *j*, as a tradeoff between the value of synergistic integrability versus the risky prospect of taking advantage of the other system’s increased openness; and (c) hypothetical assessment of system actor *i*’s expected outcomes from choosing to open or close their system (rows) contingent on actor *j*’s strategy (columns).

We consider keeping the system closed as the status quo strategy since it would not require an actor to de-constrain and expand the capabilities of their system beyond its boundaries to interact with others. In this context, pursuing synergistic integrability would be an alternative course of action—see Fig 11(a). We justify these assumptions by arguing that strategic decisions have an intended direction and are irreversible [37]. We also disregard potential halfway or “mixed” contingency plans, assuming that any decision to deviate from the status quo can be construed as a technical variation of the same alternative strategy. Thus, we focus on the incentives that affect a system actor’s decision to abandon an already in-place or taken-for-granted strategy in favor of a presumably riskier technological alternative whose potential depends on whether other actors adopt it.

#### The strategic tradeoffs: A volunteer’s dilemma

We are interested in assessing the value utility of each system actor based on how their strategic decisions would intersect. The possible strategic outcomes considered are described in Fig 11(b). We estimate each actor’s expected value using the following three rules:

1. If no system actors open their systems (*s* = *φ*), the defective integration will result in disappointing long-term outcomes for everyone (a “peril”).
2. No actor implementing an open system (*s*_*i*_ = *ψ*_*i*_) benefits significantly from increased integrability when others do the same (a “compromise”).
3. An actor who maintains a closed system (*s*_*i*_ = *φ*_*i*_) can potentially take advantage of the partial integrability resulting from others opening their systems (an “opportunity”).

Under these circumstances, and without any other actors involved, Irene and Jamie cannot take advantage of increased integrability at the same time. Focusing on Irene’s point of view, maximizing value would require them to keep the system closed while Jamie opens theirs. If Jamie refuses, the best alternative for Irene is to adopt an open approach. It is worth noting that the chance of peril may also result from a coordination failure between subsystems due to technical or operational reasons.

We summarize the expectations of any of the two actors in Fig 11(c) and assume they are (ordinally) symmetric, as their preferences depend only on the combination of strategies, not on who makes each decision. Assigning values to each outcome turns this problem into a normal-form game. The compromise of opening the system is more preferred than the peril of a failed integration but less preferred than the opportunity of taking advantage of other open systems; thus, we set “peril” = −1, “compromise” = 0, and “opportunity” = + 1. This is equivalent to a *volunteer’s dilemma*, where “volunteering” and “free-riding” are the alternative and the status quo strategies, respectively. We can model the payoff function for this game using Eq. (A.6) in Section A.4 in the S1 Appendix:

$$\begin{array}{c} U_{i}\left(s_{i},s_{\;J},s_{K}\right)=\{\begin{array}{cc} 0 & \text{if}\;s_{i}=\psi_{i}\\ +1 & \text{if}\;s_{i}=\varphi_{i}\;\wedge \;s_{\;J}=\psi_{\;J}\\ -1 & \text{otherwise}, \end{array} \end{array}$$ (22)

With two actors, there is only one value of structural fear and one value of structural greed, the former is negative and congruent with the difference between the value of “peril” and the value of “compromise”, and the latter is positive and proportional to the value of “opportunity” minus the value of “compromise.” So, it exhibits pure coexistence dynamics.

#### Potential impact of increasing the number of actors

Assume the involvement of a third actor named Kumar, who has been tasked to oversee safety and communications in the larger urban transit ecosystem and whose actions are critical to achieving synergistic integrability. Like Irene and Jamie, Kumar is presumed to be operationally and managerially independent in implementing an open or a closed system; however, Irene and Jamie may not be aware of Kumar’s exact level of autonomy. For example, Irene may believe that a governmental agency supersedes Kumar’s authority in whole or part and that whatever Kumar can do has already been defined. From Irene’s standpoint:

(a) Kumar may *never* open their system—a plausible assumption considering the prioritization of security, reliability, data privacy, and regulatory compliance.
(b) Kumar may *surely* open the safety and communications system for scalability, adaptiveness, and transparency.

In the context of the volunteer’s dilemma, it can be inferred that if Irene decides to maintain a closed system, it will likely result in favorable outcomes in scenario (b). However, if Kumar chooses not to pursue integrability, the risk of peril is greater in the event of (a). Notice that, in scenarios (a) and (b), it could be Jamie, not Kumar, the one presumed to have fixed their strategy. Also, it is possible that

(c) Jamie and Kumar align their strategies, simultaneously opening or closing their systems,

which, as in scenario (b), would make the closed system strategy riskier. If we assign the indices *i*, *j*, and *k* to the three actors, we can list the following five player-reduced binary games from the standpoint of actor *i*:

(1) actor *j*’s strategy may be uncertain, but actor *k* will surely maintain the status quo;
(2) actor *j*’s strategy may be uncertain, but actor *k* will surely choose the alternative;
(3) actor *k*’s strategy may be uncertain, but actor *j* will surely maintain the status quo;
(4) actor *k*’s strategy may be uncertain, but actor *j* will surely choose the alternative;
(5) both *j*’s and *k*’s strategies may be uncertain, but they will surely align their actions.

**Fig 12:**
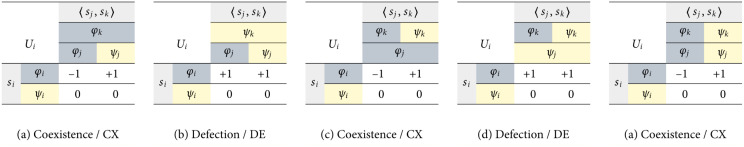
Type I player-reduced binary games that would be observed by standpoint player *i* in a 3 × 2 volunteer’s dilemma per Eq (22).

Fig 12 lists each of these games. Three of them exhibit coexistence dynamics and the remaining two exhibit defection dynamics. Specifically, using Eq. (A.7) in Section A.4 in the S1 Appendix, we obtain the strategic hindrance

$${\text{H}}_{i}^{\left(3\right)}\left(\varphi ,\psi \right)=\frac{1}{2}{\left[\begin{array}{ccccc} -1 & +1 & -1 & +1 & -1\\ +1 & +1 & +1 & +1 & +1 \end{array}\right]}^{\text{T}}.$$

If we add a fourth actor, the ratio of coexistence to defection becomes 7 to 12. With five actors, it becomes 15 to 50. The incidence of defection dynamics on each player’s strategic hindrance grows at a higher rate than the incidence of coexistence dynamics as *n* grows; more specifically,

$$\frac{\text{Number}\;\text{of}\;\text{CX}\;\text{pairs}}{\text{Number}\;\text{of}\;\text{DE}\;\text{pairs}}=\frac{2^{n-1}-1}{3^{n-1}-2^{n}+1}.$$

**Fig 13:**
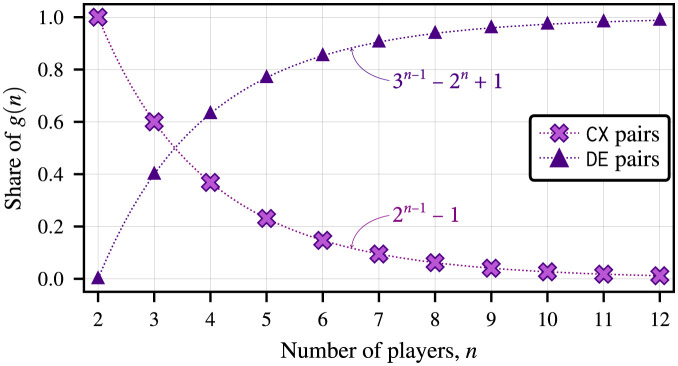
Evolution of the strategy dynamics in *n*-player volunteer’s dilemma as a proportion of the number of 〈*F*_*i*_, *G*_*i*_〉 pairs in an individual strategic hindrance space that fall on a specific domain to the total number of type I player-reduced games, *g*(*n*). The incidence of defection dynamics in the volunteer’s dilemma becomes more significant as *n* grows, while the proportion of type I player-reduced games with coexistence dynamics decreases.

We plot the evolution of coexistence and defection dynamics in the *n*-player volunteer’s dilemma in Fig 13 as the share of the total number of type I player-reduced binary games, *g*(*n*), that fall into each domain as the number of actors increases. In the context of our urban transit system example, we can expect the progress of integrability across the ecosystem to become stagnant after one actor (if any) has broken away from the status quo and implemented an open system (one of *n* possible PNE), assuming the actors are “economically rational.” However, if more subsystems were to be integrated, the increasing predominance of defection dynamics would lead to a decreasing likelihood of any system actor volunteering and opening their subsystems. This quantitative insight into the evolution of defection in the volunteer’s dilemma game is reminiscent of the prevalence of the diffusion of responsibility and bystander effect phenomena observed in real-world settings [38].

A key takeaway of this example is how crucial the efficient coordination of actions among stakeholders and systems is for optimizing resource allocation and reaching greater collective performance. For instance, if we wanted to incentivize systems-of-systems integrability in this hypothetical setting, one potential approach is to organize interconnected subsystems into small clusters to minimize the risk of coordination failure. In real-world systems-of-systems, we expect actors’ strategic tradeoffs and information to be asymmetrical and evolving, requiring the design of mechanisms to align incentives and optimize the allocation of resources. As engineering systems become more complex, managing integration and reducing incompatibility among diverse subsystems becomes increasingly challenging. Tackling these challenges will enable the management of interdependent subsystems in a harmonious way throughout each stage of the design process, ultimately leading to the creation of efficient and effective systems-of-systems that benefit society as a whole.

## 4 Discussion of assumptions and limitations

The formulation proposed in this paper takes inspiration from existing subjectivistic and epistemic approaches to game theory [39, 40], focusing less on the players’ *knowledge* and more on their *beliefs*. This worldview is present, in particular, in the computation of values of structural fear and greed for every player-reduced normal-form game—grouping one or more actors as one player (set J) and setting the strategies of any remaining player (set K) as “fixed”. We can use the latter to represent actors in the context of engineering systems whose actions have presumably been “optimized”. For example, in the example of an urban transit system in Section 3.3, a designer who believes that at least one other system actor is bound to invest in integrability might decide to take advantage of the latter. Here, the actor attempts to complete information about the game despite lacking “common” or “objective” knowledge about the likelihood of a specific combination of other players’ strategies. While the strategic hindrance space allows a standpoint player to become better informed of such combinations and understand how they are distributed across the four strategy dynamical domains, the weight that the actor assigns to each player-reduced game will still depend on their understanding of the other players and the context in which individual and collective decisions are being made.

Another instance of incompleteness of information that could be modeled as a player-reduced normal-form game is when a player is uncertain about whether the rest of the players are acting as individual agents or as a collective [41]. For example, in the El Farol bar game in Eq. (A.12) in S1 Appendix, there are players who might never go to the bar because they believe everyone else is going (by coincidence or by collusion), so it would already be 2/3 full by the time they arrive—in a dynamic context, this could lead to a “belief-distorted” Nash equilibria, as discussed by Wiszniewska-Matyszkiel [42]. For such player, going to El Farol bar is like playing a chicken game (low fear, high greed); but for those who are not so pessimistic about the bar being crowded, the game is called harmony (low fear, low greed).

Studying strategy selection in *n*-player binary normal-form games in terms of individual deviations losses is not original of this work. Examples of similar approaches can be found in Güth and Kalfoken’s [43] theory of resistance avoidance and Selten’s [34] theory of risk dominance in bipolar games. These approaches focus on measuring the likeliness that an individual remains at (or deviates towards) an equilibrium point; both works then prescribe objective ways in which such “resistances” or “diagonal probabilities” could be aggregated. Studying equilibria in player-reduced normal-form games from a subjectivistic point of view is not a new concept either. For instance, in Kalai and Lehrer’s [44] model of subjective games, players in a large game environment would restrict themselves to assessing only the portion of the game they know (viz. an “environment response function”) and approaching a subjective Nash equilibrium that might coincide with an objective equilibrium regardless of how optimistic or pessimistic their beliefs are about the “outsiders” to their subjective games. Similarly, Battigalli and Guaitoli [45] drop the “common prior assumption” (the premise of the existence of a collectively known probability distribution across the players’ strategies [46]) and favor a model of “steady-state equilibria” where an individual bases their actions on conjectures about others’ strategies and is unable (or even apathetic) to fact check.

Despite its subjectivistic nature, the proposed framework shares some similarities with existing objective and normative approaches to characterizing *n*-player games. In yet another work on equilibria selection, Güth [47] assesses the applicability of requiring consistency and non-emptiness of equilibria when choosing a solution concept. The analysis, which builds on previous work by Peleg and Tijs [48] and Norde et al. [49], questions the robustness of a solution concept (whether Nash equilibrium or not) when all players are considered and when some of the players are “gone” and their inclinations might not be revealed. On one hand, compared to Güth’s analysis, the structural fear and greed spaces provide a visual means of understanding how robust a collective strategy is to unilateral deviations, namely, by checking if those fear and greed spaces fall inside regions of single PNE like the harmony and defection strategy dynamical domains. On the other hand, the proposed framework also provides insights into how consistent a collective strategy outcome remains across all possible player-reduced games by estimating how much the players’ structural fear and greed spaces overlap. Both framework qualities do not constitute a solution concept themselves but could prove helpful when assessing incentive mechanisms to guide strategic behaviors toward desired outcomes.

A significant limitation of our framework is its focus on one-shot normal-form games where players are limited to only two pure individual strategies, and these actions are assumed to be irreversible once taken. It does not account for the possibility of mixed strategies, where players can adopt a probabilistic combination of pure strategies, which would add a layer of versatility to the model. Moreover, the assumption of irreversible actions may only sometimes be accurate in real-world engineering systems. Going back to the urban transit system example, the strategic decisions involved, while initially appearing irreversible, may be subject to change as the systems development process evolves. Feedback from the public, changing urban dynamics, unforeseen route modifications, and integration of new transportation modes, could lead to adjustments to the original “game”. Despite these limitations, our framework can serve as a supplementary early approach to evaluate the long-term implications of strategic decisions in engineering systems. It also allows researchers and practitioners to revise and reevaluate the landscape of strategic decisions and tradeoffs throughout the design process, accommodating the complexities and evolving nature of such engineering systems projects. Including mixed strategies and the possibility of reversible actions in future iterations of this framework could enhance its applicability and accuracy in representing the dynamics of strategic decision-making in complex engineering systems.

## 5 Conclusion and opportunities for future work

This paper formalizes the concept of fear and greed to describe two dimensions of strategic hindrances in strategic games with more than two players. Coming up from an engineering systems background, we discuss a motivating example highlighting the importance of strategic decisions in our field before introducing our proposed approach to studying strategic decision-making processes while capturing the complexities of incentives and relationships between actors. Building on non-cooperative game theory literature, we present a method to dissect large normal-form games, repackage them into player-reduced instances from each player’s standpoint, and embrace a subjectivistic lens to scan each player’s overall strategic stability. Our framework helps us visualize the shape and scope of the strategy dynamics that influence the players’ strategic behavior. This framework can help engineering systems researchers and practitioners identify and mitigate the structural forces that hinder the alignment of individual actions toward reaching and sustaining collective harmony.

The proposed framework can be extended to applications in cognitive engineering, particularly in enhancing the understanding and management of strategic decision-making in engineering systems design. By modeling strategic interactions as simplified games, this framework allows for a more explicit conceptualization of the human and socio-technical elements that underlie strategic decision-making processes. In cognitive engineering, where the emphasis is on aligning human cognitive capabilities with the design and operation of complex systems, our approach seeks to simplify the intricate decision-making landscape and make it more accessible and understandable from a human cognitive perspective. The mathematical rigor of the framework provides a structured way to analyze and interpret strategic interactions and reconcile human intuition with quantitative analysis. This aspect is particularly beneficial in enhancing strategic decision-support systems to augment human cognition. Our framework can be integrated into such tools and facilitate the presentation of complex strategic scenarios in a more digestible format, allowing for a more comprehensive evaluation of options and their potential consequences.

In exploring the intricacies of strategic decision-making in the context of collective systems, we encounter several challenges that require further investigation. These challenges present us with valuable opportunities to enrich our understanding and develop frameworks that help sustain collaboration among operationally and managerially independent actors in the design of engineering systems. This framework could serve as a foundation for developing practical incentive mechanisms and network interventions in future work. This direction aims to facilitate the emergence of more favorable strategy dynamics within engineering systems. By understanding the nuances of individual and collective strategic behaviors, researchers and practitioners can identify opportunities to guide actors toward more mutually beneficial and sustainable strategies subtly. For instance, tailored incentives can help align individual motivations with broader system goals, thus encouraging cooperative behaviors that positively contribute to the overall health and efficiency of the system.

Network interventions can also help rectify interactions and dependencies between blocs and coalitions of actors whose strategies significantly influence the strategic hindrance of a specific decision-maker. These interventions could include adjusting communication channels and redefining linked incentives to enhance collaboration and reduce friction in decision-making processes. Applying and refining these approaches within the framework’s structure can help steer engineering systems toward more efficient and harmonious operations. Extending this framework in such practical directions can unlock a more grounded and realistic understanding of how strategic decisions play out in complex engineering environments. Ultimately, this could lead to more resilient and adaptive engineering systems capable of navigating the challenges of evolving socio-technical landscapes. We hope this line of work helps unlock new strategies and frameworks that propel engineering systems design to tackle increasingly complex socio-technical challenges.

## Supporting information

S1 Appendix (PDF)
