# Supplementary material for: The strategy dynamics of collective systems: Underlying hindrances beyond two-actor coordination
Source: PLoS One. 2024 Apr 1;19(4):e0301394. doi: 10.1371/journal.pone.0301394 (PMC10984537; doi:10.1371/journal.pone.0301394)
Supplement: S1 Appendix — (PDF) [file pone.0301394.s001.pdf]

Appendix

A.1 Pure-strategy Nash equilibria in normal-form games

Definition A.1 *Let $\mathcal{G}_{\mathcal{N}} = (\mathcal{N}, (\mathcal{S}_i)_{i\in\mathcal{N}}, (U_i)_{i\in\mathcal{N}})$ be a normal-form game. A collective strategy $s^\star = \langle s_i^\star, s_{-i}^\star\rangle \in \mathcal{S}$ is a pure-strategy Nash equilibrium (PNE) if for each player $i \in \mathcal{N}$ and each $s_i \in \mathcal{S}_i$ the following inequality is satisfied:*

$$U_i(s_i^\star, s_{-i}^\star) - U_i(s_i, s_{-i}^\star) \geq 0.$$

We now provide an equivalent definition of PNE for binary normal-form games.

Definition A.2 *Let $\mathcal{G}_{\mathcal{N}}$ be a $n\times 2$ game, and let $\mathcal{S}_i = \{s_i, s_i^\star\}$. A collective strategy $s^\star = \langle s_i^\star, s_{-i}^\star\rangle \in \mathcal{S}$ is a PNE in $\mathcal{G}_{\mathcal{N}}$ if*

$$\min_{i\in\mathcal{N}} \Big[U_i(s_i^\star, s_{-i}) - U_i(s_i, s_{-i})\Big] \geq 0.$$

We can also characterize PNE in $n\times 2$ games in terms of the rescaled deviation losses ℓ_i in Definition 1. From Eq. (4), we know that

$$U_i(s_i^\star, s_{-i}) - U_i(s_i, s_{-i}^\star) \propto \ell_i(s_i^\star, s_{-i}^\star).$$

We can use this relationship to restate Definition A.2 as follows

Definition A.2a *Let $\mathcal{G}_{\mathcal{N}}$ be a $n\times 2$ game, and let $s_i, s_i^\star \in \mathcal{S}_i = \{\varphi_i, \psi_i\}$, where $s_i \neq s_i^\star$. A collective strategy $s^\star = \langle s_i^\star, s_{-i}^\star\rangle \in \mathcal{S}$ is a PNE in $\mathcal{G}_{\mathcal{N}}$ if*

$$\min_{i\in\mathcal{N}} \ell_i(s_i^\star, s_{-i}^\star) \geq 0.$$

A.2 Types and number of player-reduced binary games

Definition 2 in Section 3 describes the notion of a player-reduced binary game as an event in which a standpoint player i in the normal-form game $\mathcal{G}_{\mathcal{N}}$ makes early conjectures about the strategic behavior of a subset of players $\mathcal{K} \subset \mathcal{N} \setminus \{i\}$. We express this as

$$\mathcal{G}_{\mathcal{N}\setminus\mathcal{K}} = \Big(\mathcal{N}\setminus\mathcal{K}, (\mathcal{S}_i)_{i\in\mathcal{N}\setminus\mathcal{K}}, (U_i(\,\cdot\,, s_{\mathcal{K}}))_{i\in\mathcal{N}\setminus\mathcal{K}}\Big). \tag{13}$$

where $s_{\mathcal{K}}$ is the collective action by the players in $\mathcal{K}$. Player i would move on to making conjectures about the strategic behavior of the remaining players in the non-empty subset $\mathcal{J} = \mathcal{N}\setminus(\mathcal{K}\cup\{i\})$. We then characterize structural fear and greed in Definition 3 under two premises: 1) that the players in $\mathcal{J}$ would align their strategies, moving collectively from a status quo $s_{\mathcal{J}} = \varphi_{\mathcal{J}}$ towards the alternative $s_{\mathcal{J}} = \psi_{\mathcal{J}}$; and 2) that the actions of those in $\mathcal{K}$ would remain fixed at $s_{\mathcal{K}} = \sigma_{\mathcal{K}}$.

We refer to the first and second premises as *aligned $\mathcal{J}$-players* and *stationary $\mathcal{K}$-players*, respectively, and we label the player-reduced binary games that meet both conditions as *type I*. Relaxing the second premise allows us to elicit other types of player-reduced binary games that could be observed by player i in $\mathcal{G}_{\mathcal{N}}$. In particular, for $n > 2$, there are games Type II $\mathcal{G}_{\mathcal{N}\setminus\mathcal{K}}$ where one or more players $k \in \mathcal{K}$ whom player i expects will not commit to individual actions $s_k = \sigma_k \in \sigma_{\mathcal{K}}$. We call these player-reduced binary games *type II*, and we refer to the alternative premise associated with it as *reversing $\mathcal{K}$-players*. We respectively denote by $g(n)$ and $g^*(n)$ the numbers of possible type I and type II player-reduced games observed by a player i in $\mathcal{G}_{\mathcal{N}}$, explain their derivation, and provide multiple analytic expressions to calculate them.

Number of player-reduced binary games of type I: stationary K-players

To determine the expression for $g(n)$, we note that

- The value of $g(n)$ is also the number of ways in which we can partition set $\mathcal{N}\setminus\{i\}$ into one non-proper subset ($\mathcal{J}$) and one proper subset ($\mathcal{K}$).
- We must count all possible $\sigma_{\mathcal{K}}$ in Eq. (17) for every $\mathcal{K}\subset\mathcal{N}\setminus\{i\}$. Let $\mathcal{Q}$ be a subset of $\mathcal{K}$ and let its complement be $\mathcal{Q}^c=\mathcal{K}\setminus\mathcal{Q}$. We can use $\mathcal{Q}$ and $\mathcal{Q}^c$ to identify the players in $\mathcal{K}$ who presumably committed to φ_k and ψ_k, respectively — that is, $\sigma_{\mathcal{K}}=\langle\varphi_Q,\psi_{Q^c}\rangle$. It follows that, since either $\mathcal{Q}$ or $\mathcal{Q}^c$ can be empty, the set of possible $\sigma_{\mathcal{K}}$ has the same cardinality as $\mathcal{P}(\mathcal{K})$, the set of all subsets (or power set) of $\mathcal{K}$: $2^{|\mathcal{K}|}$.
- Every possible $\mathcal{K}$ contains between zero and $n-2$ players taken from $\mathcal{N}\setminus\{i\}$. Also, the number of $\mathcal{K}$ of the same size is the number of $|\mathcal{K}|$-combinations of elements in $\mathcal{N}\setminus\{i\}$. Then, the value of $g(n)$ can be expressed as the sum of all the products between $C(n-1,k)$ — the binomial coefficient — and the total number of possible $\sigma_{\mathcal{K}}$, which we know is $2^{|\mathcal{K}|}$.

These give us our first formula for the number of player-reduced games:

$$g(n)=|\{\mathcal{G}_{\mathcal{N}\setminus\mathcal{K}}\}|=\sum_{\mathcal{K}\subset\mathcal{N}\setminus\{i\}}|\mathcal{P}(\mathcal{K})|=\sum_{|\mathcal{K}|=0}^{n-2}2^{|\mathcal{K}|}\cdot\binom{n-1}{|\mathcal{K}|}; \tag{A.1}$$

adding and subtracting the $(n-1)$-th term gives:

$$g(n)=-2^{n-1}\cdot\cancelto{1}{\binom{n-1}{n-1}}+\sum_{|\mathcal{K}|=0}^{n-1}2^{|\mathcal{K}|}\cdot(1^{|\mathcal{K}|})\cdot\binom{n-1}{|\mathcal{K}|};$$

which by means of the binomial identity simplifies to

$$g(n)=3^{n-1}-2^{n-1},\qquad\forall n\geq 1. \tag{18}$$

Starting with $g(1)=0$ (no game), Eq (18) returns the Lucas sequence

$$0,1,5,19,65,211,665,2059,6305,19171,\ldots$$

listed as sequence `A001047` in Sloane's *Encyclopedia* [1].

The value of $g(n)$ can also be expressed in terms of Stirling numbers of the second kind, $\left\{{n \atop k}\right\}$, which count the number of ways to partition a set of n elements into k non-empty subsets, as follows [2]:

$$g(n)=2\left\{{n \atop 3}\right\}+\left\{{n \atop 2}\right\};$$

where $\left\{{n \atop 3}\right\}=\frac{1}{2}(1+3^{n-1})-2^{n-1}$ and $\left\{{n \atop 2}\right\}=2^{n-1}-1$. For instance, if $\mathcal{N}=\{i,j,k\}$,

$$\left\{{3 \atop 3}\right\}=1\qquad\because\mathcal{N}=\{i\}\cup\{j\}\cup\{k\};$$
$$\text{and}\quad\left\{{3 \atop 2}\right\}=3\qquad\because\mathcal{N}=\{i\}\cup\{j,k\}=\{i,j\}\cup\{k\}=\{i,k\}\cup\{j\}.$$

So $g(3)=2(1)+3=5$. In the context of the static games with $n>2$ treated in this work, the term $\left\{{n \atop 3}\right\}$ is associated with the number of player-reduced games where the individual strategies of at least two players other than i differ, i.e. $s_j\neq s_k$, where $j,k\in\mathcal{N}\setminus\{i\}$; and the term $\left\{{n \atop 2}\right\}$ is associated with the player-reduced games $\mathcal{G}_{\mathcal{N}\setminus\mathcal{K}}$ whose strategy profiles include the diagonal collective strategy $s_{\text{-}i}\in\{\varphi_{\text{-}i},\psi_{\text{-}i}\}$, i.e. $s_1=\cdots=s_{i-1}=s_{i+1}=\cdots=s_n$.

The directed graphs in Fig A.1 describe the player-reduced binary games with fixed $s_{\mathcal{K}}=\sigma_{\mathcal{K}}$, $\mathcal{K}\subset\mathcal{N}\setminus\{i\}$, for $2\leq n\leq 4$. The vertices represent the possible collective strategies $s_{\text{-}i}=\in\mathcal{S}_i^{n-1}$ that the standpoint player i faces. Each arc represents one player-reduced game; the source and target vertices are associated with structural fear and structural greed, respectively.

n	$\mathcal{J}$	$\mathcal{K}$	$\sigma_{\mathcal{K}}$	$\mathcal{G}_{\mathcal{N}\setminus\mathcal{K}}$
2	2	$\emptyset$	n/a	A
3	2	3	φ_3<br>ψ_3	B<br>C
	3	2	φ_2<br>ψ_2	D<br>E
	−1	$\emptyset$	n/a	F
4	2	{3,4}	$\langle\varphi_3,\varphi_4\rangle$<br>$\langle\psi_3,\varphi_4\rangle$<br>$\langle\varphi_3,\psi_4\rangle$<br>$\langle\psi_3,\psi_4\rangle$	G<br>H<br>I<br>J
	3	{2,4}	$\langle\varphi_2,\varphi_4\rangle$<br>$\langle\psi_2,\varphi_4\rangle$<br>$\langle\varphi_2,\psi_4\rangle$<br>$\langle\psi_2,\psi_4\rangle$	K<br>L<br>M<br>N
	4	{2,3}	$\langle\varphi_2,\varphi_3\rangle$<br>$\langle\psi_2,\varphi_3\rangle$<br>$\langle\varphi_2,\psi_3\rangle$<br>$\langle\psi_2,\psi_3\rangle$	O<br>P<br>Q<br>R
	{2,3}	4	φ_4<br>ψ_4	S<br>T
	{2,4}	3	φ_3<br>ψ_3	U<br>V
	{3,4}	2	φ_2<br>ψ_2	W<br>X
	−1	$\emptyset$	n/a	Y

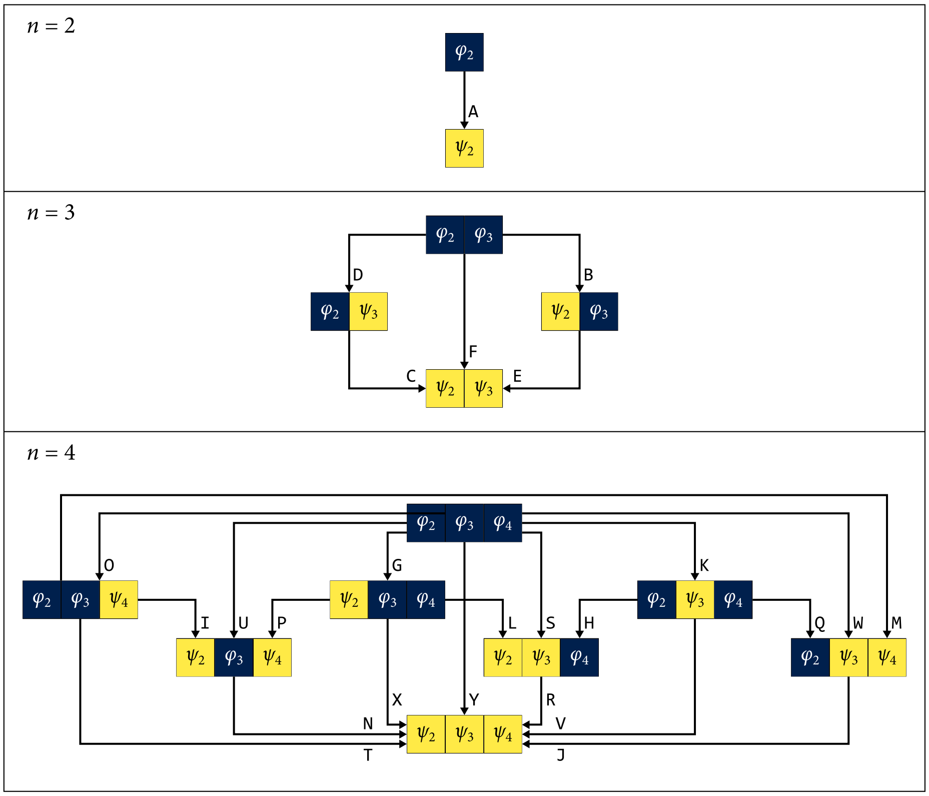

Fig A.1. Type I player-reduced normal-form games $\mathcal{G}_{\mathcal{N}\setminus\mathcal{K}}$ observed by each player in $n \times 2$ games for $2 < n < 4$. Every player-reduced game is formed by the actions of standpoint player $i = 1$, the coordinated actions of the players in set $\mathcal{J} = \mathcal{N}\setminus(\mathcal{K} \cup \{i\})$, and the presumably set in stone action $\sigma_{\mathcal{K}}$ by all players in $\mathcal{K}$. On the right, we create directed graphs made of 2^{n-1} vertices, one for every collective strategy $s_{\text{-}i} = \langle s_{\mathcal{J}}, s_{\mathcal{K}}\rangle$, and connect them according to the possible deviations under the premises of aligned $\mathcal{J}$-players and stationary $\mathcal{K}$-players. Then, each edge represents a possible player-reduced game (listed from A to Y); and the source and target vertices respectively represent the components of $s_{\text{-}i}$ in $\ell_i(s_{\text{-}i})$ needed to compute the values of F_i and G_i per Eqs. (15) and (16).

Number of player-reduced binary games of type II: reversing K-players

We derive $g^*(n)$ by analyzing the discrete geometry of all possible transitions between two different collective strategies $s_{\text{-}i}$ and $s^*_{\text{-}i}$ that can be observed by player i. Either action can be equal to any possible combinations of $n-1$ individual strategies $s_j \in \mathcal{S}_j = \{\varphi_j, \psi_j\}$. So, the total number of possible transitions is

$$|\mathcal{S}_j^{n-1} \times \mathcal{S}_j^{n-1}| - |\mathcal{S}_j^{n-1}| = (2^{n-1}) \cdot (2^{n-1}) - 2^{n-1} = 4^{n-1} - 2^{n-1}.$$

In calculating the number of type I player-reduced binary games $g(n) = 3^{n-1} - 2^{n-1}$, we assumed the collective action $s_{\mathcal{J}}$ by the aligned $\mathcal{J}$-players moved in the direction $\varphi_{\mathcal{J}} \to \psi_{\mathcal{J}}$. Asserting the premise of reversing $\mathcal{K}$-players, we note type I player-reduced binary games also extend to $s_{\mathcal{J}}$ moving in the opposite direction, namely $\psi_{\mathcal{J}} \to \varphi_{\mathcal{J}}$. This means that the total number of type II player-reduced binary games must be equal to

$$\begin{aligned} g^*(n) &= 4^{n-1} - 2^{n-1} - 2 \cdot g(n) \\ &= 4^{n-1} - 2(3^{n-1}) + 2^{n-1}. \end{aligned} \tag{A.2}$$

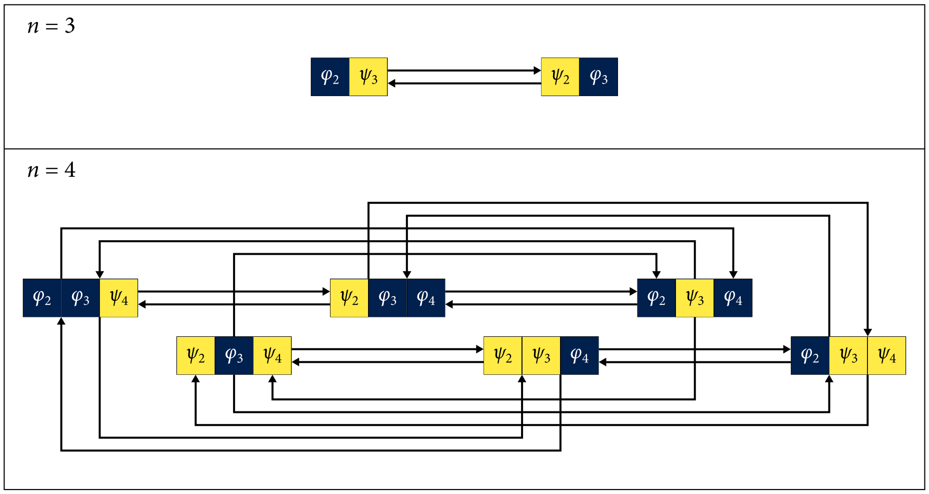

Fig A.2. Transitions between non-diagonal collective strategies representing the number of player-reduced games with anti-coordination for $n = 3$ and $n = 4$. Let $\mathcal{J} \subset \mathcal{N} \backslash \{i\}$ and $\mathcal{K} = \mathcal{N} \backslash (\mathcal{J} \cup \{i\})$; and let Q be a subset of $\mathcal{K}$, with complement $Q^c = \mathcal{K} \backslash Q$. As all players in $\mathcal{J}$ deviate from $\varphi_{\mathcal{J}} \to \psi_{\mathcal{J}}$, all players in Q deviate from $\psi_Q \to \varphi_Q$ while the players in Q^c, if any, keep their strategies fixed. The number of player-reduced games following the aforementioned description of anti-coordination is $4^{n-1} - 2(3^{n-1}) - 2^{n-1}$. This figure complements Fig A.1.

Fig A.2 shows the directed graphs describing the associations between collective strategies that result in type II player-reduced games and that complement the type I games presented in Fig A.1 for $n = 3$ and $n = 4$ — note that $g^*(2) = 0$ consistent with $\mathcal{K} = \emptyset$. Combined, the total number of player-reduced binary games with stationary and reversing $\mathcal{K}$-players assuming the aligned $\mathcal{J}$-players move from $\varphi_{\mathcal{J}}$ towards $\to \psi_{\mathcal{J}}$ is

$$g(n) + g^*(n) = 4^{n-1} - 3^{n-1}.$$

We can also count $g(n)$ and $g^*(n)$ by breaking apart the transition between any two collective strategies into the moves by each player and labeling the number of existing intersecting collective actions transitioning in the same direction. This approach is demonstrated using square grids in Fig A.3 for $n \in [2\,..\,4]$. The result shows the number $g(n)$ emerge as the number of states in the Hanoi graph analog of the Sierpiński triangle — from the Tower of Hanoi puzzle with 3 towers and $n-1$ disks — minus the 2^{n-1} states along the diagonal $s_j = s_j^*$; while the number $g^*(n)$, counting all type II player-reduced binary games, emerge as two times the number of even values in Pascal's triangle up to row 2^{n-1}.

A.3 Distribution of deviation losses in strategic hindrance spaces

We characterize the incidence of the rescaled deviation losses $\ell_i^*(s_{-i})$ on $\mathbf{H}_i^{(n)}(\varphi, \psi)$, player i's strategic hindrance in Definition 4, across observed type I player-reduced games. Let us set

- $\mathcal{N}_\varphi$ and $\mathcal{N}_\psi$ as the subsets of players in $\mathcal{N}$ that play φ and ψ, respectively;
- $n_\varphi = |\mathcal{N}_\varphi|$ and $n_\psi = |\mathcal{N}_\psi|$ as the numbers of players that adopt such strategies; and
- $n_{\varphi\backslash i} = |\mathcal{N}_\varphi \setminus \{i\}|$ and $n_{\psi\backslash i} = |\mathcal{N}_\psi \setminus \{i\}|$, so $n_{\varphi\backslash i} + n_{\psi\backslash i} = n - 1$.

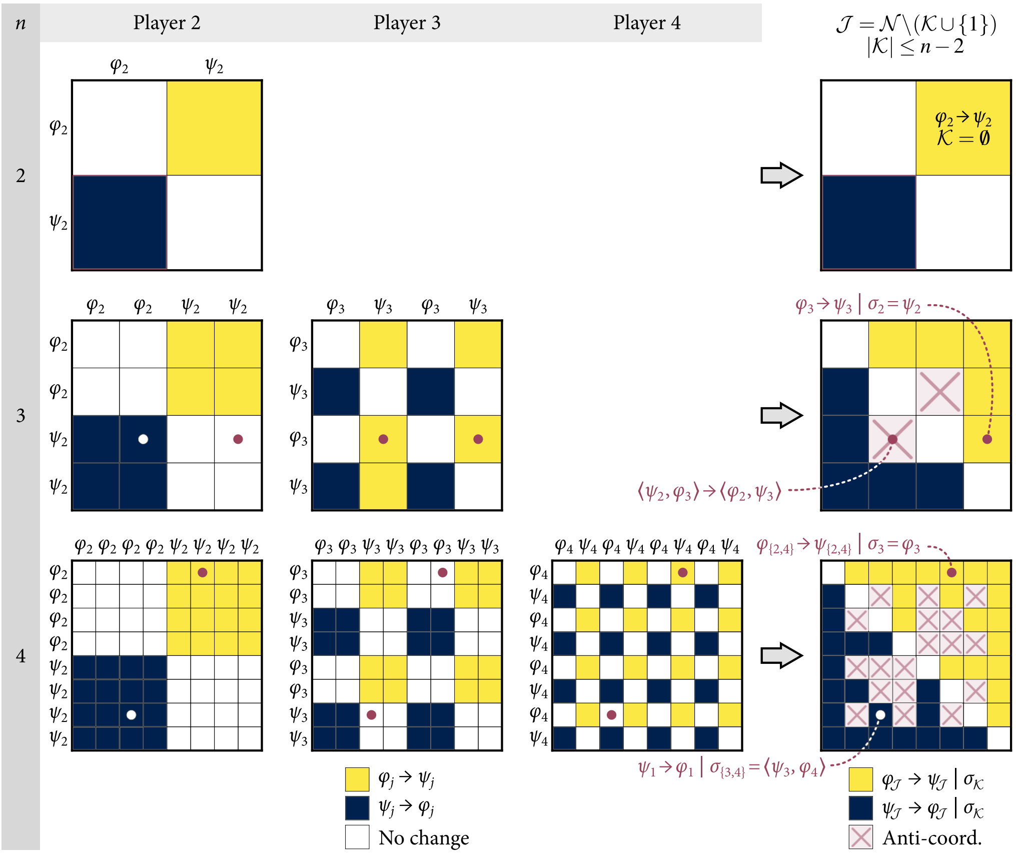

Fig A.3. Alternative approach to counting the number of player-reduced binary games observed by one player (viz. $i = 1$) in an $n \times 2$ game, for $n \in [2\,..\,4]$. Each grid on the rightmost column shows all possible transitions from s_{-i} and s^*_{-i}; these grids are broken apart into individual transitions from s_j and $s^*_j \in \mathcal{S}_j$ — $\varphi_j \to \psi_j$ in yellow/light gray and $\psi_j \to \varphi_j$ in dark blue/gray, going from row to column; and either null transitions of stationary $\mathcal{K}$-players — blank cells, if any, with "no changes" — or reversed transition of reversing $\mathcal{K}$-players — marked with an $\times$.

Recall that $\mathcal{J} = \mathcal{N} \setminus (\mathcal{K} \cup \{i\})$ and $|\mathcal{J}| \in [1\,..\,n-1]$. Replacing $k = |\mathcal{K}| = n - 1 - |\mathcal{J}|$ in Eq. (A.1), gives us an expression for $g(n)$ in terms of $\mathcal{J}$:

$$g(n) = \sum_{|\mathcal{J}|=1}^{n-1} 2^{n-1-|\mathcal{J}|} \cdot \binom{n-1}{n-1-|\mathcal{J}|} = \sum_{|\mathcal{J}|=1}^{n-1} (2^{|\mathcal{J}|} - 1) \cdot \binom{n-1}{|\mathcal{J}|} = \sum_{\mathcal{J} \subseteq \mathcal{N} \setminus \{i\}} |\mathcal{P}^+(\mathcal{J})|, \tag{A.3}$$

where $\mathcal{P}^+(\mathcal{J})$ is the set of all non-empty subsets of $\mathcal{J}$. From the definition of strategic hindrance in Eq. (17), we know $\dim_{\mathbb{R}}(\langle F_i \rangle) = \dim_{\mathbb{R}}(\langle G_i \rangle) = g(n)$ and all players in $\mathcal{J}$ would coordinate on either $\varphi_{\mathcal{J}}$ or $\psi_{\mathcal{J}}$. Let $|\mathcal{J}| = n_{\varphi \setminus i}$ if $s_{\mathcal{J}} = \varphi_{\mathcal{J}}$ and $|\mathcal{J}| = n_{\psi \setminus i}$ if $s_{\mathcal{J}} = \psi_{\mathcal{J}}$. We can write

$$\begin{aligned} g(n) &= \frac{1}{2} \left[\dim_{\mathbb{R}} (\langle F_i \rangle) + \dim_{\mathbb{R}} (\langle G_i \rangle) \right] \\ &= \frac{1}{2} \left[\sum_{n_{\varphi\setminus i}=0}^{n-1} (2^{n_{\varphi\setminus i}} - 1) \cdot \binom{n-1}{n_{\varphi\setminus i}} \right] + \frac{1}{2} \left[\sum_{n_{\psi\setminus i}=0}^{n-1} (2^{n_{\psi\setminus i}} - 1) \cdot \binom{n-1}{n_{\psi\setminus i}} \right] \end{aligned} \tag{A.4}$$

after adding and subtracting the 0-th term to the summation (without affecting the interpretation of $g(n)$ using Eq. (A.3), as $2^0 - 1 = 0$).

$\gamma_{\times C(n-1,k)}$		k											
n	$g(n)$	0	1	2	3	4	5	6	7	8	9	10	11
1	0	$0_{\times 1}$											
2	1	$1_{\times 1}$	$1_{\times 1}$										
3	5	$3_{\times 1}$	$2_{\times 2}$	$3_{\times 1}$									
4	19	$7_{\times 1}$	$4_{\times 3}$	$4_{\times 3}$	$7_{\times 1}$								
5	65	$15_{\times 1}$	$8_{\times 4}$	$6_{\times 6}$	$8_{\times 4}$	$15_{\times 1}$							
6	211	$31_{\times 1}$	$16_{\times 5}$	$10_{\times 10}$	$10_{\times 10}$	$16_{\times 5}$	$31_{\times 1}$						
7	665	$63_{\times 1}$	$32_{\times 6}$	$18_{\times 15}$	$14_{\times 20}$	$18_{\times 15}$	$32_{\times 6}$	$63_{\times 1}$					
8	2,059	$127_{\times 1}$	$64_{\times 7}$	$34_{\times 21}$	$22_{\times 35}$	$22_{\times 35}$	$34_{\times 21}$	$64_{\times 7}$	$127_{\times 1}$				
9	6,305	$255_{\times 1}$	$128_{\times 8}$	$66_{\times 28}$	$38_{\times 56}$	$30_{\times 70}$	$38_{\times 56}$	$66_{\times 28}$	$128_{\times 8}$	$255_{\times 1}$			
10	19,171	$511_{\times 1}$	$256_{\times 9}$	$130_{\times 36}$	$70_{\times 84}$	$46_{\times 126}$	$46_{\times 126}$	$70_{\times 84}$	$130_{\times 36}$	$256_{\times 9}$	$511_{\times 1}$		
11	58,025	$1{,}023_{\times 1}$	$512_{\times 10}$	$258_{\times 45}$	$134_{\times 120}$	$78_{\times 210}$	$62_{\times 252}$	$78_{\times 210}$	$134_{\times 120}$	$258_{\times 45}$	$512_{\times 10}$	$1{,}023_{\times 1}$	
12	175,099	$2{,}047_{\times 1}$	$1{,}024_{\times 11}$	$514_{\times 55}$	$262_{\times 165}$	$142_{\times 330}$	$94_{\times 462}$	$94_{\times 462}$	$142_{\times 330}$	$262_{\times 165}$	$514_{\times 55}$	$1{,}024_{\times 11}$	$2{,}047_{\times 1}$

Fig A.4. Number of reduced-player games, $g(n)$ from Eq. (A.1), and values of $\gamma(k, n-1-k)$, $k \in \{n_{\varphi\setminus i}, n_{\psi\setminus i}\}$, from Eq. (A.5) observed by each player in games with up to $n = 12$ players. The subscripts denote the occurrences of each γ and are equal to $C(n-1,k)$. For every integer $n \geq 1$, the sum of the $\gamma(k, n-1-k) \cdot C(n-1,k)$ products equal $2g(n)$ per Eq. (20). For $n = 3$, notice that the deviation losses $\ell_i^*(\varphi_2, \psi_3)$ and $\ell_i^*(\psi_2, \varphi_3)$ would be weighted twice ($k = 1$); this is consistent with the directed graph representation of the player-reduced games for $n = 3$, in Fig A.1, where both vertices $\langle \varphi_2, \psi_3 \rangle$ and $\langle \psi_2, \varphi_3 \rangle$ are once a source and once a target.

Redefining $g(n)$ in terms of $n_{\varphi\setminus i}$ and $n_{\psi\setminus i}$ allows us to count the number of player-reduced games where a specific number of players in $\mathcal{N} \setminus \{i\}$ select one strategy or the other. Adding one to $n_{\varphi\setminus i}$ means subtracting one from $n_{\psi\setminus i}$, and vice versa. Let $n_{\varphi\setminus i} = k$ and $n_{\psi\setminus i} = n-1-k$; we can rewrite Eq. (A.4) as

$$g(n) = \frac{1}{2}\left[\sum_{k=0}^{n-1}(2^k - 1)\cdot\binom{n-1}{k}\right] + \frac{1}{2}\left[\sum_{k=0}^{n-1}(2^{n-1-k} - 1)\cdot\binom{n-1}{n-1-k}\right]$$

$$g(n) = \frac{1}{2}\left[\sum_{k=0}^{n-1}\gamma(k, n-1-k)\cdot C(n-1,k)\right] \tag{20}$$

where $C(n-1,k) = \binom{n-1}{k} = \binom{n-1}{n-1-k}$ and the function

$$\gamma(k, n-1-k) = 2^k + 2^{n-1-k} - 2, \tag{A.5}$$

or

$$\gamma(n_{\varphi\setminus i}, n_{\psi\setminus i}) = 2^{n_{\varphi\setminus i}} + 2^{n_{\psi\setminus i}} - 2, \tag{19}$$

counts how many entries of each individual strategic hindrance space of size $g(n) \times 2$ are equal to $\ell_i^*(s_{\text{-}i})$ based solely on the number of players in $\mathcal{N} \setminus \{i\}$ that play φ and those that play ψ. Values of $\gamma(k, n-1-k)$ and their occurrences for games with up to $n = 12$ players are provided in Fig A.4. The triangular arrays arising from the computation of $\gamma(n-1-k, k)$ and the product $\gamma(n-1-k, k)\cdot C(n-1, k)$ can be found in Sloane's *Encyclopedia* [1] under the catalog numbers A350770 and A350771, respectively.

A.4 Strategy dynamics of 3×2 social dilemma games

This section calculates the strategic hindrance spaces of nine social dilemmas in normal form and $n = 3$. The strategic hindrance of every player in each game are the same; thus, their strategy dynamics can be considered symmetrical. All of the strategic hindrance spaces are compiled in Fig A.5.

Volunteer's dilemma

Each player can either volunteer for the common good (ψ) or do nothing and possibly benefit from the contributions of others (φ) — helping maintain the status quo. Player i's payoff in this game is defined as

$$U_i(s_i, s_{\mathcal{J}}, s_{\mathcal{K}}) = \begin{cases} 0 & \text{if } s_i = \psi_i \\ a & \text{if } s_i = \varphi_i \wedge s_{\mathcal{J}} = \psi_{\mathcal{J}} \\ -b & \text{otherwise,} \end{cases} \tag{A.6}$$

where a and b are both positive real numbers. Each player in the volunteer's dilemma in Eq. (A.6) has one *perfectly limited strategy*, viz. ψ_i, which yields constant $U_i(\psi_i, s_{\text{-}i})$ for any $s_{\text{-}i} \in \mathcal{S}_i^{n-1}$ (i.e. the payoff of volunteering does not depend on whether others also volunteer or not) restricting interactive effects [3]. There are exactly n PNE in this game, each of them equal to $s^\star = \langle \psi_i, \varphi_{\text{-}i} \rangle$ for every $i \in \mathcal{N}$: every time one and only one player has volunteered.

Incentives modeled after a volunteer's dilemma can lead to maintaining the status quo becoming the dominant strategy. After one actor has chosen a cooperative strategy, such as investing in integrability in the context of the urban transit system, others, acting economically rational, may decide to seize such opportunity and take advantage of the volunteer's contribution without reciprocating. As all actors would prefer to keep their systems closed to avoid the disadvantages of being the only one to open their system, integration could fail due to collective inaction. Defection dynamics are anticipated to emerge and dominate the conflict as the number of players increases.

Using Eqs. (14)–(16) to obtain the rescaled deviation losses in this game for every player $i \in \mathcal{N}$ — with the help of Table 1:

$$\begin{aligned}
\ell_i^*(\varphi_j, \varphi_k) &= A_i^{-1} \cdot \Big(U_i(\varphi_i, \varphi_j, \varphi_k) - U_i(\psi_i, \varphi_j, \varphi_k)\Big) = \big(-b - 0\big)/A_i = -b/A_i, \\
\ell_i^*(\varphi_j, \psi_k) &= A_i^{-1} \cdot \Big(U_i(\varphi_i, \varphi_j, \psi_k) - U_i(\psi_i, \varphi_j, \psi_k)\Big) = \big(\;\; a - 0\big)/A_i = \;\;\, a/A_i, \\
\ell_i^*(\psi_j, \varphi_k) &= A_i^{-1} \cdot \Big(U_i(\varphi_i, \psi_j, \varphi_k) - U_i(\psi_i, \psi_j, \varphi_k)\Big) = \big(\;\; a - 0\big)/A_i = \;\;\, a/A_i, \\
\ell_i^*(\psi_j, \psi_k) &= A_i^{-1} \cdot \Big(U_i(\varphi_i, \psi_j, \psi_k) - U_i(\psi_i, \psi_j, \psi_k)\Big) = \big(\;\; a - 0\big)/A_i = \;\;\, a/A_i.
\end{aligned}$$

where $A_i = \max U_i - \min U_i = a + b$. Using Eq. (21), the strategic hindrance is:

$$\mathbf{H}_i^{(3)}(\varphi, \psi) = \frac{1}{a+b} \begin{bmatrix} -b & +a & -b & +a & -b \\ +a & +a & +a & +a & +a \end{bmatrix}^{\mathrm{T}}. \tag{A.7}$$

Three of the $g(3) = 5$ player-reduced binary games exhibit coexistence while the remaining two exhibit defection. Fig A.5 shows the strategic hindrance space of the 3×2 volunteer's dilemma for $a = 1$ and $b = 2$.

Diner's dilemma

Three graduate students go for lunch and agree to split the bill equally before ordering. When the menu arrives, they choose individually between ordering the combo plate with extra protein (φ) or just a fresh empanada (ψ). The difference in cost between dishes, c (in utility units), is

believed to be greater than the difference in satisfaction, b; that is $0 < b < c$. However, they might be tempted to order the more satisfying combo plate if they suspect everyone else will order empanadas, implying that $b > c/n$ — which guarantees $U_i(\varphi_i, s_{\text{-}i}) > U_i(\psi_i, s_{\text{-}i})$, signaling pure defection dynamics. Let $\mathcal{N}_\varphi \subseteq \mathcal{N}$ be the subset of diners ordering the combo plate, $n_\varphi = |\mathcal{N}_\varphi|$, and let $n_{\varphi \setminus i} = |\mathcal{N}_\varphi \setminus \{i\}|$; player i's payoff is modeled as

$$U_i(s_i, s_{\text{-}i}) = \begin{cases} b - n_\varphi \left(\dfrac{c}{n}\right) & \text{if } s_i = \varphi_i \\ -n_{\varphi\setminus i} \left(\dfrac{c}{n}\right) & \text{otherwise.} \end{cases} \tag{A.8}$$

From Eq. (A.8), we notice that the difference in individual payoff between choosing $s_i = \varphi_i$ and choosing $s_i = \psi_i$ for a given $s_{\text{-}i} \in \mathcal{S}_i^{n-1}$ always equals $\max U_i = b - c/n > 0$. This game has a single PNE (viz. φ) for any $n \geq 2$. The 3-player diner's dilemma in Fig A.5 uses $b = 1$ and $c = 2$. Using Eqs. (14)–(16) and Table 1:

$$\begin{aligned}
\ell_i^*(\varphi_j, \varphi_k) &= A_i^{-1} \cdot \left[\left(1 - 3 \cdot \tfrac{2}{3}\right) - \left(-2 \cdot \tfrac{2}{3}\right) \right] = +(1/3)/A_i, \\
\ell_i^*(\varphi_j, \psi_k) &= A_i^{-1} \cdot \left[\left(1 - 2 \cdot \tfrac{2}{3}\right) - \left(-1 \cdot \tfrac{2}{3}\right) \right] = +(1/3)/A_i, \\
\ell_i^*(\psi_j, \varphi_k) &= A_i^{-1} \cdot \left[\left(1 - 2 \cdot \tfrac{2}{3}\right) - \left(-1 \cdot \tfrac{2}{3}\right) \right] = +(1/3)/A_i, \\
\ell_i^*(\psi_j, \psi_k) &= A_i^{-1} \cdot \left[\left(1 - 1 \cdot \tfrac{2}{3}\right) - \left(\ \ 0 \cdot \tfrac{2}{3}\right) \right] = +(1/3)/A_i;
\end{aligned}$$

where $A_i = \max U_i - \min U_i = b + c - 2 \cdot c/n = 5/3$. All rescaled deviation losses are signed positive; ordering the combo plate instead of just a pastry is always the dominant strategy. The strategic hindrance is, using Eq. (21):

$$\mathbf{H}_i^{(3)}(\varphi, \psi) = \frac{1}{5/3} \begin{bmatrix} +\frac{1}{3} & +\frac{1}{3} & +\frac{1}{3} & +\frac{1}{3} & +\frac{1}{3} \\ +\frac{1}{3} & +\frac{1}{3} & +\frac{1}{3} & +\frac{1}{3} & +\frac{1}{3} \end{bmatrix}^{\mathrm{T}} = \frac{1}{5} \begin{bmatrix} +1 \\ +1 \end{bmatrix}^{\mathrm{T}}_{\times 5}.$$

Glance and Huberman [4] introduced the diner's dilemma as a potential extension of the PD game to study the emergence of cooperation in settings with more than two players. A player-reduced binary game is a classical 2×2 PD game if

$$U_i(\varphi_i, \psi_{\mathcal{J}}, \sigma_{\mathcal{K}}) > U_i(\psi_i, \psi_{\mathcal{J}}, \sigma_{\mathcal{K}}) > U_i(\varphi_i, \varphi_{\mathcal{J}}, \sigma_{\mathcal{K}}) > U_i(\psi_i, \varphi_{\mathcal{J}}, \sigma_{\mathcal{K}}), \tag{A.9}$$

which is equivalent to the relationship $T > R > P > S$ from Section 2.2 and Fig 1. Setting

$$\frac{1}{n} < \frac{b}{c} < \frac{n+1}{2 \cdot n}$$

guarantees that all player-reduced games in a diner's dilemma are classical 2×2 PD games.

Public goods game (PGG)

The public goods game (PGG) with linear variable contributions is defined by

$$U_i(s_i, s_{\text{-}i}) = \begin{cases} 1 + n_{\psi\setminus i} \left(\dfrac{a_i}{n}\right) & \text{if } s_i = \varphi_i \\ n_\psi \left(\dfrac{a_i}{n}\right) & \text{otherwise,} \end{cases} \tag{A.10}$$

where $n_\psi = |\mathcal{N}_\psi|$, $n_{\psi\setminus i} = |\mathcal{N}_\psi \setminus \{i\}|$, and $\mathcal{N}_\psi \subseteq \mathcal{N}$ is the subset of players that contribute one token to the public pool (ψ). Not contributing to the public pool is represented by strategy φ_i. Factor $a_i > 0$ multiplies the tokens in the pool before dividing them evenly among all players. Similar to Eq. (A.8), the image of Eq. (A.10) contains only two values per player, meaning the absolute individual deviation losses are constant (and equal to $1 - a_i/n$), and each player

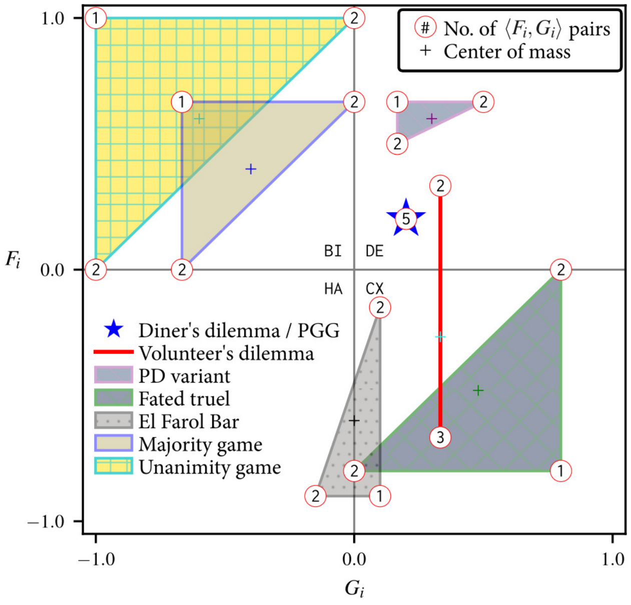

Fig A.5. A player fear and greed value space in seven 3×2 social dilemmas normal-form games. Some fear and greed value pairs are repeated.

observes dominance strategy dynamics depending on the value of a_i. For $n = 3$ and setting $a_i = 2$, Eq. (21) gives the strategic hindrance space

$$\mathbf{H}_i^{(3)}(\varphi,\psi) = \frac{1}{5/3}\begin{bmatrix} +\frac{1}{3} & +\frac{1}{3} & +\frac{1}{3} & +\frac{1}{3} & +\frac{1}{3} \\ +\frac{1}{3} & +\frac{1}{3} & +\frac{1}{3} & +\frac{1}{3} & +\frac{1}{3} \end{bmatrix}^{\mathrm{T}} = \frac{1}{5}\begin{bmatrix} +1 \\ +1 \end{bmatrix}^{\mathrm{T}}_{\times 5},$$

where $A_i = \max U_i - \min U_i = 1 + a_i - 2\cdot a_i/n = 5/3$, which exhibits the same pure defection dynamics as those of the diner's dilemma example above and whose strategic hindrance is depicted in Fig A.5 using Eq. (A.8).

Similar to the diner's dilemma, we can turn every player-reduced game in a PGG into a classical 2×2 PD game if we set, $\forall i \in \mathcal{N}$:

$$\frac{n}{2} < a_i < n,$$

which ensures that every level of mutual cooperation between two or more players will always yield a greater utility than mutual defection, yet guarantee that the temptation to defect unilaterally remains higher, satisfying Eq. (A.9). Comparing Eqs. (A.8) and (A.10), we can define any diner's dilemma into a PGG by calculating

$$a_i = \frac{n-1}{2\cdot(b/c)+1-(3/n)}.$$

For values of a_i greater than n, the PGG yields harmony dynamics. Whenever $a_i \neq n$ for every $i \in \mathcal{N}$ and $n \geq 2$, the PGG has only one PNE, with each $s_i^\star$ equal to either φ_i if $\operatorname{sgn}(a-n) = -1$ or ψ_i if $\operatorname{sgn}(a-n) = +1$ — where $\operatorname{sgn} : \mathbb{R} \mapsto \mathbb{Z}$ is the signum function which returns 1 if $x > 0$, -1 if $x < 0$, and 0 if $x = 0$. If $a_i = n$, the game is that of indifference (as $n_\psi = 1 + n_{\psi\setminus i}$), and $\mathcal{S}^\star$ is trivially equal to 2^n.

A prisoner's dilemma variant

Here, we introduce an alternative version of a PD game with increased incentives for defection. Prosecutors separately promise three alleged co-conspirators in a crime a lighter treatment if they snitch on their associates (φ) instead of remaining silent (ψ). All three suspects will face a minimum sentence if they keep quiet. If some snitch while others refuse to talk, the tipsters will be acquitted, while the quiet ones will face a combined sentence of c times the minimum. But if they all confess, they will all face b times the minimum sentence. Let $1 < b < c$; player i's payoff in this game is defined as

$$U_i(s_i, s_{\mathcal{J}}, s_{\mathcal{K}}) = \begin{cases} 0 & \text{if } s_i = \varphi_i \wedge s_{\mathcal{J}} = \psi_{\mathcal{J}} \\ -1 & \text{if } \langle s_i, s_{\mathcal{J}}, s_{\mathcal{K}} \rangle = \psi \\ -b & \text{if } \langle s_i, s_{\mathcal{J}}, s_{\mathcal{K}} \rangle = \varphi \\ -c/n_{\psi} & \text{otherwise.} \end{cases}$$

The rescaled deviation losses in the 3×2 version of this game are

$$\begin{aligned}
\ell_i^*(\varphi_j, \varphi_k) &= A_i^{-1} \cdot \Big(U_i(\varphi_i, \varphi_j, \varphi_k) - U_i(\psi_i, \varphi_j, \varphi_k)\Big) = \big(-b + c \quad\big)/A_i = (c-b)/A_i, \\
\ell_i^*(\varphi_j, \psi_k) &= A_i^{-1} \cdot \Big(U_i(\varphi_i, \varphi_j, \psi_k) - U_i(\psi_i, \varphi_j, \psi_k)\Big) = \big(\ 0 + c/2\big)/A_i = +(c/2)/A_i, \\
\ell_i^*(\psi_j, \varphi_k) &= A_i^{-1} \cdot \Big(U_i(\varphi_i, \psi_j, \varphi_k) - U_i(\psi_i, \psi_j, \varphi_k)\Big) = \big(\ 0 + c/2\big)/A_i = +(c/2)/A_i, \\
\ell_i^*(\psi_j, \psi_k) &= A_i^{-1} \cdot \Big(U_i(\varphi_i, \psi_j, \psi_k) - U_i(\psi_i, \psi_j, \psi_k)\Big) = \big(\ 0 + 1 \quad\big)/A_i = \quad +1/A_i.
\end{aligned}$$

where $A_i = c$. Fig A.5 shows the individual strategic hindrance in a three-person prisoner's dilemma, with $b = 2$ and $c = 6$, calculated via Eq. (21):

$$\begin{aligned}
\mathbf{H}_i^{(3)}(\varphi, \psi) &= \frac{1}{c} \begin{bmatrix} c-b & c/2 & c-b & c/2 & c-b \\ c/2 & +1 & c/2 & +1 & +1 \end{bmatrix}^{\mathrm{T}} \\
&= \frac{1}{6} \begin{bmatrix} +4 & +3 & +4 & +3 & +4 \\ +3 & +1 & +3 & +1 & +1 \end{bmatrix}^{\mathrm{T}}.
\end{aligned}$$

This take on the n-player prisoner's dilemma game is a simplification of Weil's [5] description with a stronger condition on the payoff dominance of defection modeled after the original, classical interpretation for two actors by Merrill Flood and Melvin Dresher, and contextualized by Albert W. Tucker [6]. Condition $b < c$ guarantees that unanimous defection (φ) is always the only PNE and that it is suboptimal regardless of the number of players. And setting $b > 1$ guarantees that unanimous cooperation (ψ) is always preferred over φ. However, in contrast with the diner's dilemma and the PGG — in which every player-reduced game can be turned into a classical 2×2 PD game — the reward of staying silent in this model is lower than the punishment from defecting any time at least one suspect confesses. These characteristics also differ from alternative generalizations of the prisoner's dilemma such as those in the works by Goehring and Kahan [7] and Diekmann [8] that reduce the problem to an n-player volunteer's dilemma, which has n PNE rather than only one.

Fated truel

Consider three mutual paintball adversaries, Blondie, Angel Eyes, and Tuco, each with one paintball left, holding their markers at point-blank. Each of them must decide which of the other two rivals to fire at, the one on their left (φ_i) or the one on their right (ψ_i). To make it out clean, a standpoint player must first coincide with a second player on targetting their remaining rival and pray for the latter to target the second player. When two players target the same rival, they

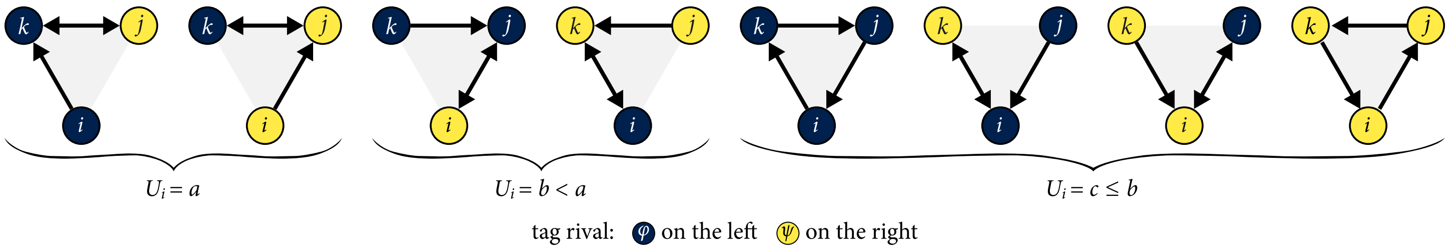

Fig A.6. Possible outcomes and individual payoffs in a fated truel game (player i's standpoint) per Eq. (A.11). Each arrow points at the players' targets.

are anti-coordinating their strategies in opposite directions to shooting each other. Specifically, two consecutive players Tuco (i) and Blondie ($i+1$), the former on the left and the latter on the right, must play φ_i and ψ_{i+1} to shoot Angel Eyes ($i-1$). Angel Eyes might still manage to mark one of them, albeit with less effectiveness. For Tuco to come out of the standoff clean, Angel Eyes must target Blondie by playing (φ_{i-1}). Let a be the expected payoff for making it out clean, b the expected payoff for firing at the same target as another player with the risk of being hit by that target, and c the expected payoff of being shot at; it follows that $a > b \geq c \in \mathbb{R}$. Player i's payoff contingent on actions by the players on their left ($i-1$) and right ($i+1$) is defined as

$$U_i(s_i, s_{i+1}, s_{i-1}) = \begin{cases} a & \text{if } s_{i+1} = \psi_{i+1} \wedge s_{i-1} = \varphi_{i-1} \\ b & \text{if } s_i \neq s_{i+1} = s_{i-1} \\ c & \text{otherwise.} \end{cases} \tag{A.11}$$

The eight possible outcomes in this game are depicted in Fig A.6. Two possible outcomes could see all players defeated in the standoff: φ (everyone targets their rival on their right) and ψ (their rival on their left). The generic individual strategic hindrance in a fated truel game is provided in Fig A.5 using $a = 2$, $b = 1$, and $c = -3$. The deviation losses in this game are:

$$\begin{aligned} \ell_i^*(\varphi_{i+1}, \varphi_{i-1}) &= A_i^{-1} \cdot [\,c - b\,] = A_i^{-1} \cdot [-3 - \quad 1\,] = -4/A_i \\ \ell_i^*(\varphi_{i+1}, \psi_{i-1}) &= A_i^{-1} \cdot [\,c - c\,] = A_i^{-1} \cdot [-3 - (-3)] = \quad 0 \\ \ell_i^*(\psi_{i+1}, \varphi_{i-1}) &= A_i^{-1} \cdot [\,a - a\,] = A_i^{-1} \cdot [\;\; 2 - \quad 2\,] = \quad 0 \\ \ell_i^*(\psi_{i+1}, \psi_{i-1}) &= A_i^{-1} \cdot [\,b - c\,] = A_i^{-1} \cdot [\;\; 1 - (-3)] = +4/A_i \end{aligned}$$

While $A_i = a - c = 2 - (-3) = 5$. The space of fear and greed values is:

$$\mathbf{H}_i^{(3)}(\varphi, \psi) = \frac{1}{5} \begin{bmatrix} -4 & 0 & -4 & 0 & -4 \\ 0 & +4 & 0 & +4 & +4 \end{bmatrix}^{\mathrm{T}}.$$

Since the payoff that player i would obtain from choosing φ_i or ψ_i depends not only on the strategies of the other players but also their identities (i.e. indices or positions), the fated truel cannot be considered a symmetric game in the classical sense, even though the resulting strategy dynamics are symmetrical. Also, notice that the fated truel game exhibits 2×2 indifference dynamics anytime $b = c$ (all values of structural fear and greed are zero) resulting in $\mathcal{S}^\star = \mathcal{S}$. When $a > b > c$, this game exhibits pure 2×2 coexistence and $\mathcal{S}^\star = \mathcal{S} \setminus \{\varphi, \psi\}$.

El Farol bar

Three groups of friends decide independently whether to have fun at the small and only bar in Santa Fé (ψ), unaware of how crowded it is. Neither group wants to be at a bar packed beyond

its limits; if they find the bar to be more than 2/3 full, they will regret not having stayed (at someone's) home (φ). So, although they would love to meet another group there, they prefer not all groups to show up, which requires anti-coordination. For any $a > b \geq 0$, player i's payoff in this game is defined as

$$U_i(s_i, s_j, s_k) = \begin{cases} 0 & \text{if } \langle s_i, s_j, s_k \rangle = \psi \\ a & \text{if } s_i = \psi_i \wedge s_{\text{-}i} = \varphi_{\text{-}i} \\ b & \text{if } s_i = \psi_i \wedge s_j \neq s_k \\ c & \text{otherwise.} \end{cases} \tag{A.12}$$

For $n = 3$, this game has 3 PNE each of them equal to $s^\star = \langle \varphi_i, \psi_{\text{-}i} \rangle$ for every $i \in \mathcal{N}$. In Fig A.5, $a = 100\%$ fun, $b = 25\%$ fun, and $c = 10\%$ fun. The individual strategic hindrance is:

$$\mathbf{H}_i^{(3)}(\varphi, \psi) = \frac{1}{100} \begin{bmatrix} -90 & -15 & -90 & -15 & -90 \\ -15 & +10 & -15 & +10 & +10 \end{bmatrix}^{\mathrm{T}}.$$

Simple majority game

A player only benefits when they are one of the $\lceil n/2 \rceil$ or more members of $\mathcal{N}$ who choose the same strategy (φ or ψ). Those who are part of a minority receive nothing. When n is even, a stalemate could result in penalties for all players. They payoff function is given by

$$U_i(s_i, s_j, s_k) = \begin{cases} 1/n_\varphi & \text{if } s_i = \varphi_i \wedge n_\varphi > n/2 \\ 1/n_\psi & \text{if } s_i = \psi_i \wedge n_\psi > n/2 \\ -c & \text{if } n_\varphi = n_\psi = n/2 \\ 0 & \text{otherwise,} \end{cases} \tag{A.13}$$

where $c \geq 0$ is the associated penalty for failing to form a simple majority. The set of PNE in this game is $\mathcal{S}^\star = \{\varphi, \psi\}$. For $n = 3$ and $c = 0$, the individual strategic hindrance is:

$$\mathbf{H}_i^{(3)}(\varphi, \psi) = \frac{1}{1/2} \begin{bmatrix} +\frac{1}{3} & 0 & +\frac{1}{3} & 0 & +\frac{1}{3} \\ 0 & -\frac{1}{3} & 0 & -\frac{1}{3} & -\frac{1}{3} \end{bmatrix}^{\mathrm{T}}.$$

Matching pennies with more than 2 players

Matching pennies are zero-sum variants of simple majority games. In these games, the sum of the benefits received by the players who form a majority must equal the sum of the absolute losses that minority players incur. The payoff function in a symmetric version of these games is

$$U_i(s_i, s_j, s_k) = \begin{cases} 0 & \text{if } n_\varphi \in \{0, n/2, n\} \\ -n_\psi/n_\varphi & \text{if } s_i = \varphi_i \wedge n_\varphi \in [1 \mathinner{..} n/2) \\ -n_\varphi/n_\psi & \text{if } s_i = \psi_i \wedge n_\psi \in [1 \mathinner{..} n/2) \\ 1 & \text{otherwise,} \end{cases} \tag{A.14}$$

The individual payoff matrix for a three-player version of this game is provided in Figure A.7. The corresponding strategic hindrance space is

$$\mathbf{H}_i^{(3)}(\varphi, \psi) = \frac{1}{3} \begin{bmatrix} +2 & 0 & +2 & 0 & +2 \\ 0 & -2 & 0 & -2 & -2 \end{bmatrix}^{\mathrm{T}},$$

same as that of the three-player simple majority game in Fig A.5 using Eq. (A.13) with $c = 0$. While $\mathcal{S}^\star = \{\varphi, \psi\}$ for $n > 2$, the classical 2-player game version of this game in Eq. (12), characterized by $\operatorname{sgn}(U_i(s_i, s_j)) = -\operatorname{sgn}(U_j(s_j, s_i)) \neq 0$, has no PNE.

$U_i(s_i, s_j, s_k)$	$s_k = \varphi_k$		$s_k = \psi_k$	
	$s_j = \varphi_j$	$s_j = \psi_j$	$s_j = \varphi_j$	$s_j = \psi_j$
$s_i = \varphi_i$	0	1	1	−2
$s_i = \psi_i$	−2	1	1	0
$U_i(\varphi_i, s_j, s_k) - U_i(\psi_i, s_j, s_k)$	2	0	0	−2

Fig A.7. Individual payoff matrix in a three-player matching pennies game, a zero-sum variant of a majority game. The peak-to-peak payoff amplitude is $A_i = 3$. The assigning of φ or ψ as "heads" or "tails" can be done arbitrarily. The resulting structural fear and greed value space is the same as the one for the majority game in Fig A.5.

Unanimity game

All players in $\mathcal{N}$ must choose the same strategy (φ or ψ) to reap a benefit $b > 0$:

$$U_i(s_i, s_{\text{-}i}) = \begin{cases} b & \text{if } s_1 = \cdots = s_{i-1} = s_i = s_{i+1} = \cdots = s_n \\ 0 & \text{otherwise.} \end{cases}$$

For $\mathcal{N} = \{i, j, k\}$, the deviation losses in this game are: $\ell_i^*(\varphi_{\text{-}i}) = -\ell_i^*(\psi_{\text{-}i}) = b/A_i = 1$ and $\ell_i^*(\varphi_j, \psi_k) = \ell_i^*(\psi_j, \varphi_k) = 0$. The individual strategic hindrance is:

$$\mathbf{H}_i^{(3)}(\varphi, \psi) = \frac{1}{b} \begin{bmatrix} +b & 0 & +b & 0 & +b \\ 0 & -b & 0 & -b & -b \end{bmatrix}^{\mathrm{T}}.$$

All type I player-reduced binary games in a $n{\times}2$ unanimity game are $2{\times}2$ bistability games. Under these dynamics, stable rational collective action requires that every player belongs to a (sub-) coalition with at least one other player willing to choose the same strategy. With $n = 3$, only two of such coalitions can be formed: $\langle\varphi_1, \varphi_2, \varphi_3\rangle$ and $\langle\psi_1, \psi_2, \psi_3\rangle$. With $n = 4$, there are eight ways in which rational players would align their strategies: two instances in which all players choose the same strategy (i.e. φ and ψ) plus six instances in which two of them play φ_i and the other two play ψ_i. We can obtain the number of PNE as $|\mathcal{S}^\star| = \max\{2, 2^n - 2n\}$ for $n \geq 2$, which adds the two collective diagonal strategies, φ and ψ, and every possible binary sequence of length $n = n_\varphi + n_\psi$ where $n_\varphi > 1$ and $n_\psi > 1$ (sequence `A052515` in Sloane's *Encyclopedia* [1]).

A $n{\times}2$ game with no PNE

After characterizing the relationship between deviation losses, the emergence of strategy dynamics, and equilibria conditions in several classical $n{\times}2$ social dilemmas, we conclude with a formula that recursively constructs a binary game with no PNE for $n > 2$. Building on the bimatrix game in Eq. (11) and Definition A.2a, the following formula allocates a balanced ternary payoff vector $U \in \{-1,\ 0,\ +1\}^n$ to every $s \in \mathcal{S}$ such that $\min_{i \in \mathcal{N}} \ell_i(s_i, s_{\text{-}i}) < 0$; by assigning a strict cyclic order to $\mathcal{N}$, e.g. $[1, 2, \ldots, n-1, n] \Rightarrow [i, i+1, \ldots, i-2, i-1]$, the perfectly limited individual payoff function

$$U_i(s_i, s_{\mathcal{J}}, s_{\mathcal{K}}) = \begin{cases} 0 & \text{if } s_i = \varphi_i \\ +1 & \text{if } s_{\mathcal{J}} = \langle\varphi_{i+1}, \varphi_{i-1}\rangle \vee s_{\mathcal{J}} = \langle\psi_{i+1}, \varphi_{i+2}\rangle \\ -1 & \text{otherwise,} \end{cases} \tag{A.15}$$

where $i \in [1\,..\,n]$, guarantees that there is at least one player in $\mathcal{N}$ and no more than $n-1$ that would be better off deviating away from any $s \in \mathcal{S}$. For instance, if $n = 3$, the rescaled deviation

losses for player $i = 1$ are

$$\begin{aligned}
\ell_i^*(\varphi_{i+1},\varphi_{i-1}) &= A_i^{-1}\cdot\Big(U_i(\varphi_i,\varphi_{i+1},\varphi_{i-1}) - U_i(\psi_i,\varphi_{i+1},\varphi_{i-1})\Big) = \big(\ 0-1\big)/A_i = -b/A_i,\\
\ell_i^*(\varphi_{i+1},\psi_{i-1}) &= A_i^{-1}\cdot\Big(U_i(\varphi_i,\varphi_{i+1},\psi_{i-1}) - U_i(\psi_i,\varphi_{i+1},\psi_{i-1})\Big) = \big(\ 0+1\big)/A_i = +b/A_i,\\
\ell_i^*(\psi_{i+1},\varphi_{i-1}) &= A_i^{-1}\cdot\Big(U_i(\varphi_i,\psi_{i+1},\varphi_{i-1}) - U_i(\psi_i,\psi_{i+1},\varphi_{i-1})\Big) = \big(\ 0-1\big)/A_i = -b/A_i,\\
\ell_i^*(\psi_{i+1},\psi_{i-1}) &= A_i^{-1}\cdot\Big(U_i(\varphi_i,\psi_{i+1},\psi_{i-1}) - U_i(\psi_i,\psi_{i+1},\psi_{i-1})\Big) = \big(\ 0+1\big)/A_i = +b/A_i;
\end{aligned}$$

where $A_i = 2$. The individual strategic hindrance is:

$$\begin{aligned}
\mathbf{H}_i^{(3)}(\varphi,\psi) &= \frac{1}{A_i}\begin{bmatrix} \ell_i^*(\varphi_{i+1},\varphi_{i-1}) & \ell_i^*(\varphi_{i+1},\psi_{i-1}) & \ell_i^*(\varphi_{i+1},\varphi_{i-1}) & \ell_i^*(\psi_{i+1},\varphi_{i-1}) & \ell_i^*(\varphi_{i+1},\varphi_{i-1}) \\ \ell_i^*(\psi_{i+1},\varphi_{i-1}) & \ell_i^*(\psi_{i+1},\psi_{i-1}) & \ell_i^*(\varphi_{i+1},\psi_{i-1}) & \ell_i^*(\psi_{i+1},\psi_{i-1}) & \ell_i^*(\psi_{i+1},\psi_{i-1}) \end{bmatrix}^{\mathrm{T}}\\
&= \frac{1}{2}\begin{bmatrix} -1 & +1 & -1 & -1 & -1 \\ -1 & +1 & +1 & +1 & +1 \end{bmatrix}^{\mathrm{T}}.
\end{aligned}$$

References

1. OEIS Foundation Inc. The On-Line Encyclopedia of Integer Sequences; 2024. Available from: `https://oeis.org/`.

2. La Haye R. Binary Relations on the Ppower Set of an n-Element Set. J Integer Seq. 2009;12(09.2.6).

3. Grogan PT, Ho K, Golkar A, de Weck OL. Multi-actor Value Modeling for Federated Systems. IEEE Syst J. 2018;12(2):1193–1202. doi:10.1109/JSYST.2016.2626981.

4. Glance NS, Huberman BA. The Dynamics of Social Dilemmas. Sci Am. 1994;270(3):76–81.

5. Weil RL. The N-person Prisoner's Dilemma: Some Theory and a Computer-oriented Approach. Behav Sci. 1966;11(3):227–234. doi:10.1002/bs.3830110310.

6. Poundstone W. Prisoner's Dilemma. New York, NY, United States: Anchor Books; 1992.

7. Goehring DJ, Kahan JP. The Uniform n-Person Prisoner's Dilemma Game: Construction and Test of an Index of Cooperation. J Confl Resolut. 1976;20(1):111–128. doi:10.1177/002200277602000104.

8. Diekmann A. Volunteer's Dilemma. J Confl Resolut. 1985;29(4):605–610. doi:10.1177/0022002785029004003.
